# Metagenomic methylation patterns resolve bacterial genomes of unusual size and structural complexity

**DOI:** 10.1038/s41396-022-01242-7

**Published:** 2022-04-22

**Authors:** Elizabeth G. Wilbanks, Hugo Doré, Meredith H. Ashby, Cheryl Heiner, Richard J. Roberts, Jonathan A. Eisen

**Affiliations:** 1 Department of Ecology, Evolution and Marine Biology, University of California, Santa Barbara, CA USA; 2 Pacific Biosciences, Menlo Park, CA USA; 3 New England Biolabs, Ipswich, MA USA; 4 Department of Ecology and Evolution, University of California, Davis, CA USA

**Keywords:** Evolution, Microbiology, Molecular biology

## Abstract

The plasticity of bacterial and archaeal genomes makes examining their ecological and evolutionary dynamics both exciting and challenging. The same mechanisms that enable rapid genomic change and adaptation confound current approaches for recovering complete genomes from metagenomes. Here, we use strain-specific patterns of DNA methylation to resolve complex bacterial genomes from long-read metagenomic data of a marine microbial consortium, the “pink berries” of the Sippewissett Marsh (USA). Unique combinations of restriction-modification (RM) systems encoded by the bacteria produced distinctive methylation profiles that were used to accurately bin and classify metagenomic sequences. Using this approach, we finished the largest and most complex circularized bacterial genome ever recovered from a metagenome (7.9 Mb with >600 transposons), the finished genome of *Thiohalocapsa* sp. PB-PSB1 the dominant bacteria in the consortia. From genomes binned by methylation patterns, we identified instances of horizontal gene transfer between sulfur-cycling symbionts (*Thiohalocapsa* sp. PB-PSB1 and *Desulfofustis* sp. PB-SRB1), phage infection, and strain-level structural variation. We also linked the methylation patterns of each metagenome-assembled genome with encoded DNA methyltransferases and discovered new RM defense systems, including novel associations of RM systems with RNase toxins.

## Introduction

In nature, bacterial and archaeal genomes are far from the tidy, static sequence of letters in our databases. They are, quite simply, alive—with all the dynamism and complexity that we associate with life. Genomes can change substantially within the lifetime of a single cell, catalyzed by the intra- and inter-genomic shuffling of homologous recombination, mobile genetic elements, and phages [1, 2]. Unlike the gradual accumulation of point mutations, such bulk rearrangements can abruptly diversify an organism’s phenotypic traits and alter its niche [3–7]. Horizontal gene transfer (HGT), such as the acquisition of pathogenicity islands or antibiotic resistance genes from other species, is perhaps the most notorious example of recombination abruptly changing an organism’s capabilities. However, even small-scale recombination within an organism’s own genome can alter important phenotypes, such as biofilm formation regulated by excision/insertion of an insertion-sequence (IS) element [8–10]. The very same molecular features that enable rapid evolutionary change (genomic repeats, unusual sequence content and composition) also present analytical challenges, creating a disturbing blind spot in our study of microbial eco-evolutionary dynamics.

Metagenomic assembly algorithms often founder when confronted with repetitive sequences. DNA sequences generated by most commonly used high-throughput methods are too short to unambiguously resolve the correct path through these complex regions of the assembly graph [11]. From samples with co-existing strains of the same species or for organisms primed for rearrangements because of their richness in repeats such as transposons, we typically recover only genomic “shrapnel”, their recombination hotspots expunged. The highest quality metagenome-assembled genomes (MAGs) often come from the most clonal species in a community [e.g., ref. [12]], not necessarily the most abundant or ecologically important [13]. Assembly shortcomings beget further challenges, as smaller assembled sequences (contigs or scaffolds) are more difficult to correctly assign to their genomes of origin (i.e., binning).

Binning algorithms that classify assembled metagenomic sequences based on a set of shared, distinctive genome-wide signals similarly struggle with recombination hotspots and mobile elements [13]. Commonly used signals include phylogenetic profiles (sequence similarity to known organisms, e.g., ref. [14]), sequence composition (GC content or tetranucleotide frequency, e.g., refs. [15, 16]), and relative abundance (coverage variation within a sample or across samples, e.g., ref. [17]). Accurate bins draw support from multiple, concordant signals that persist across all the sequences constituting the draft genome [18, 19]. However, in the mosaic genomes of many bacteria and archaea, such genome-wide consistency does not exist. Infecting (pro)phages, mobile elements, and horizontally transferred genes all have evolutionary histories distinct from their host genome. The discord in phylogenetic and compositional profiles between these regions and the rest of the genome confounds binning algorithms relying on such signals [20]. It remains challenging to faithfully reunite those sequence fragments that once comingled within the cell. Recent advances in binning leverage information about the genome orthogonal to its sequence, such as chromosomal conformation [21, 22] or DNA methylation [23].

**Fig. 1:**
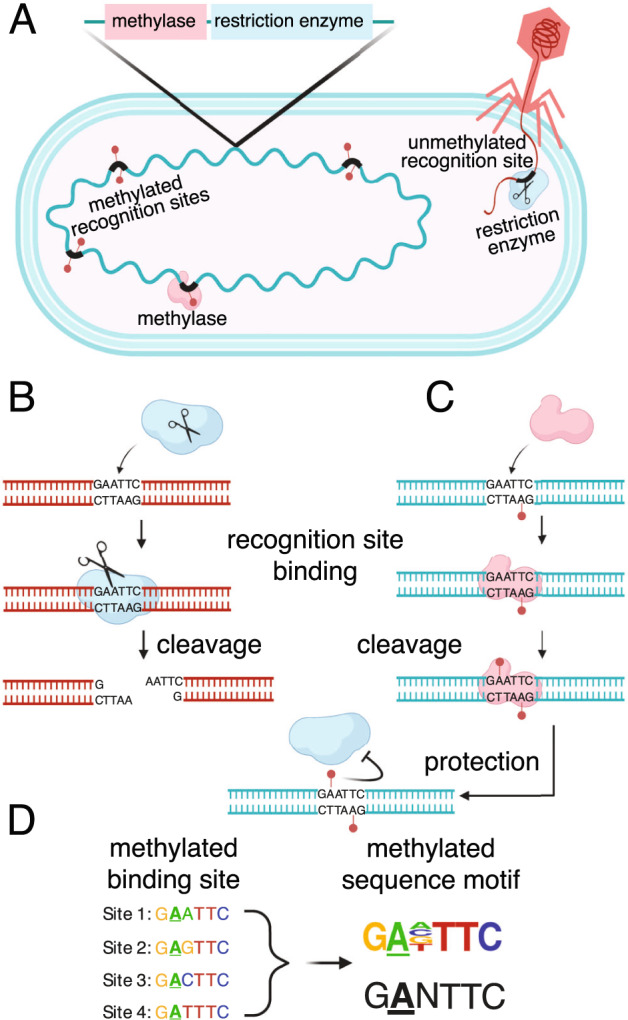
Restriction-modification (RM) systems provide bacteria and archaea with a defense against foreign DNA by discriminating self from non-self DNA based on methylation patterns. **A** RM systems, such as the Type II RM illustrated here, consist of a methyltransferase (MTase, pink) and restriction enzyme (RE, blue) that both recognize short, specific sequences in the genome (“recognition binding sites”, thick black lines). **B** Unmethylated recognition sites, such as the example shown in an infecting phage genome, are cleaved by the RE. **C** The MTase protects the host genome from degradation by the RE. The MTase binds and methylates the recognition site (which is often palindromic) at a specific base. The MTase binds and modifies hemi-methylated recognition sites, where one but not both strands are unmethylated, a characteristic that helps the cell discriminate newly replicated host DNA (hemi-methylated) from completely unmethylated foreign DNA. Methylation of both 5’ and 3’ strands in the recognition site inhibits the cognate RE, protecting that site from cleavage. **D** MTases (and their cognate REs) often tolerate variation in some positions of their recognition sites, as shown for position 3, in this example. A MTase’s binding site sequence can be discovered by analyzing the sequence context around methylated bases in the genome, and summarized by a sequence motif where the methylated base is underlined (shown here as a sequence frequency logo, top, or a consensus sequence, bottom).

In the present work, we studied the DNA methylation signals that bacteria and archaea use to discriminate their own genome from foreign DNA to overcome issues with assembling and binning complex microbial genomes from metagenomes. The most common base modifications in bacterial and archaeal genomes are made by the DNA methyltransferases (MTases), frequently associated with restriction-modification (RM) systems [24]. The restriction endonuclease of an RM system defends the host from foreign DNA by cleaving unmethylated DNA at sequence-specific recognition sites (Fig. 1). The cognate MTase methylates recognition sites in the host’s genome, thereby protecting them from restriction enzyme activity [Fig. 1C, ref. [25]]. Beyond their role in host defense, MTases have been shown to play important physiological roles, from regulating gene expression to DNA replication and repair [26].

Specific MTase recognition sites can be discovered from genome-wide surveys of DNA modification by examining the short stretches of sequence surrounding the methylated base and summarizing recurrent patterns as methylated motifs (Fig. 1D, ref. [27]). RM systems are diverse and widespread amongst bacteria and archaea, and like many defense systems, they vary greatly even between closely related species [28]. We identified strain-specific methylation patterns on metagenomic contigs from the DNA polymerase kinetics of Pacific Biosciences (PacBio) sequence data. We used this methylation information to bin and assemble bacterial genomes of unusual size and structural complexity from a microbial consortium, the “pink berries” of the Sippewissett marsh (Massachusetts, USA), macroscopic microbial aggregates for which we previously recovered complete but highly fragmented MAGs [29]. The pink berries are primarily a consortium of a purple sulfur bacterial species (PB-PSB1) and a sulfate-reducing bacterial species (PB-SRB1) that form a specific association involving direct transfer of sulfur compounds. Though the consortia’s evenness is highly skewed towards these two most abundant taxa, there also exist a diversity of other community members including marine diatoms and *Bacteroidetes* [29].

## Results

### Methylation in metagenomes: detection and clustering of sequence data

PacBio data from “pink berry” aggregates were assembled to produce 18 megabases (Mb) of sequence on 169 contigs, with an N50 of 413 kb, where the largest contig was 3.5 Mb in size (Supplementary Tables 1 and 2). This assembly recruited back 87% of the error corrected reads, indicating that it was a reasonable representation of the data. N^6^ -methyladenine (6mA) was detected on every contig (modification QV ≥ 20, i.e., *p* value ≤0.01), while N^4^ -methylcytosine (4mC) was detected on 152 out of the 169 contigs (Supplementary Fig. 1 and Supplementary Data 1). The average frequency of 6mA detections per 10 kb of contig sequence was independent of sequence depth above 40× coverage, indicating good detection sensitivity for most assembled contigs (Supplementary Fig. 2A). In contrast, 4mC modifications were both rarer and strongly correlated with sequencing depth up to ~60× coverage, which suggests decreased detection sensitivity on many contigs (Supplementary Fig. 2B).

Thirty-two sequence motifs were identified from the sequence context of these methylations by analyzing a subset of large contigs in the dataset using the SMRT Analysis workflow (Supplementary Table 3). For each of these motifs, we quantified how many times the sequence occurred on a contig and whether that sequence was methylated. Thus, each contig has a “methylation profile” composed of 32 distinct methylation metrics, quantifying the proportion of a motif’s occurrences that were methylated.

### Methylation-based clustering recovers metagenome-assembled genomes

**Fig. 2:**
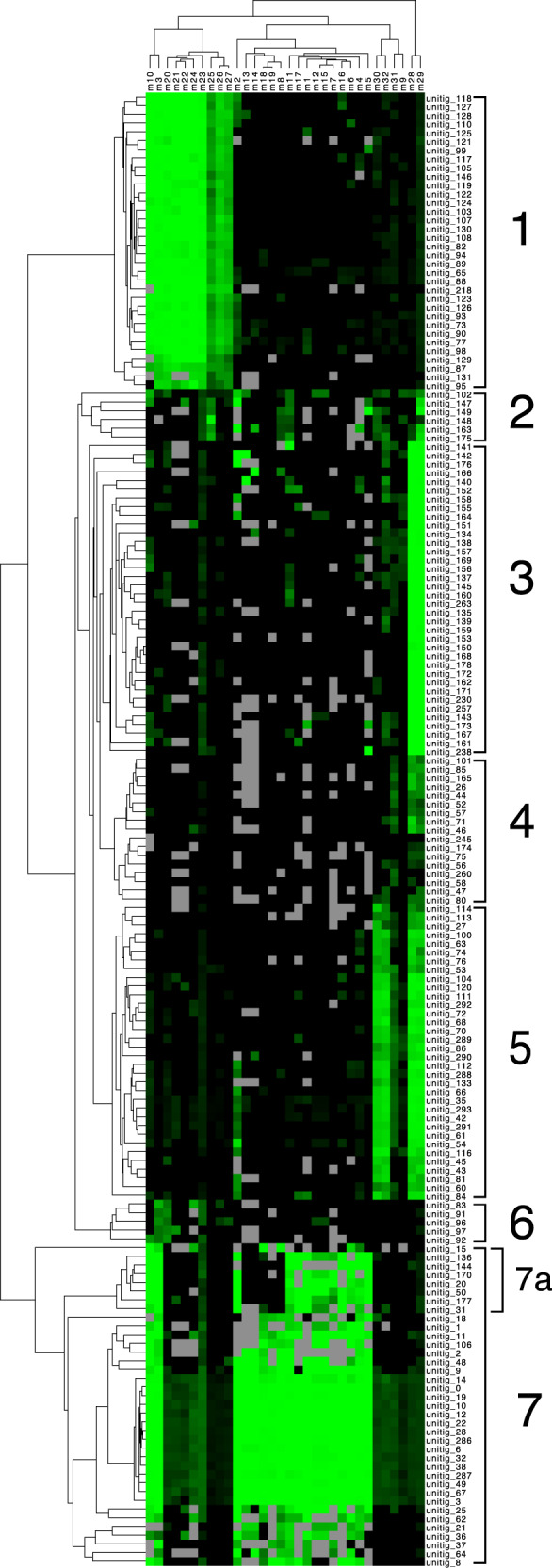
DNA methylation patterns cluster metagenomic contigs into distinct groups. The methylation status of 32 distinct sequence motifs (m1-m32, columns) is shown on every metagenomic contig (rows, unitig 1 – unitig 169). The value plotted is the percentage of motifs methylated on every contig (square root transformed); bright green color indicates a motif for which every instance on that contig was methylated (100%), and black shows motifs for which no instances were methylated on that contig (0%). When no instances of the sequence motif were observed on a contig, this is indicated as missing data (gray). Rows and columns have been hierarchically clustered using average pairwise linkage based on Euclidean distance. Manually defined methylation groups based on this clustering have been numbered 1–7. A high-resolution version of this figure with legible column and row text is provided in Supplementary Data 2.

**Fig. 3:**
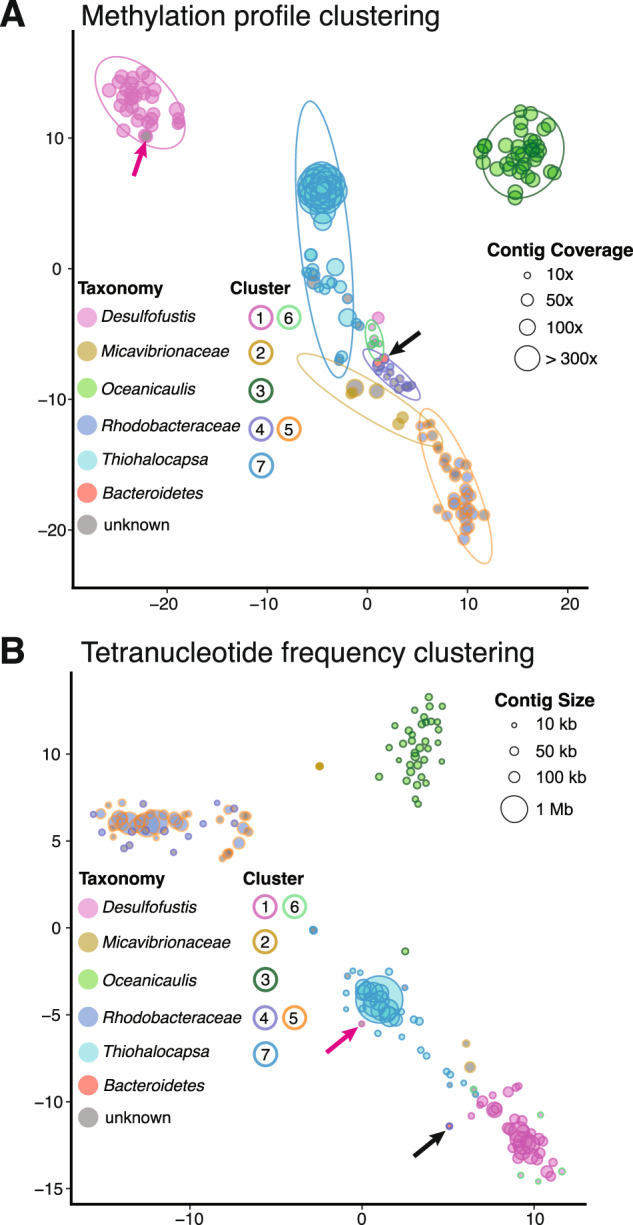
Contigs clustered by methylation profiles create taxonomically coherent bins. Similarities between contigs were visualized with t-distributed stochastic neighbor embedding (t-SNE) of either **(A**) methylation profiles or **(B)** tetranucleotide frequencies. Point size is scaled to either contig coverage (**A**) or contig length (**B**). Fill color corresponds to the taxonomic assignment and outline color represents the methylation groups defined in Fig. 2. Prediction data ellipses in (**A**) were defined for these methylation groups with the assumption that the population is a multivariate t-distribution. Black arrows indicate the three overlapping low coverage, low GC (<45%) contigs within methylation group 4 that represent contamination from the *Bacteroidetes*. Pink arrows indicate a contig that had discordant binning between methylation profiling and tetranucleotide frequency analyses.

The methylation profiles differed significantly between metagenomic contigs, and hierarchical clustering of this data revealed seven distinct groups (Fig. 2and Supplementary Data 2). These groups were recapitulated by independent clustering using t-distributed stochastic neighbor embedding (t-SNE) of the methylation profiles, and these groups represented taxonomically coherent bins of the dominant organisms in the consortia (Fig. 3A and Table 1). These methylation groups were also largely consistent with similarities in sequence composition, such as tetranucleotide frequency and GC content (Fig. 3B and Supplementary Figs. 3 and 4). Incomplete methylation-based bins (e.g., groups 2 and 3 shown in Table 1) were also validated against a set of complete, draft-quality MAGs extracted from a parallel and deeply sequenced Illumina metagenome.

**Table 1:** Summary of the seven methylation groups of metagenomic contigs defined in Fig. 2.

Group	Taxonomic assignment	Contigs	Size (bp)	Maximum length	N50	Average coverage	Maximum coverage	Minimum coverage	Completeness (%)^a^	Contamination (%)^b^	Strain heterogeneity (%)^c^
1	*Desulfofustis* PB-SRB1	34	4,050,579	605,607	231,618	51	74	28	99	0	0
2	*Micavibrionaceae*	6	159,502	75,497	15,262	50	110	24	0	0	0
3	*Oceanicaulis alexandrii*	36	615,423	33,317	18,029	64	84	47	17	0	0
4	*Rhodobacteraceae*	17	255,557	25,798	15,190	14	40	7	0	0	0
5	*Rhodobacteraceae*	34	4,521,144	784,839	219,055	33	46	15	95	2	77
6	*Desulfofustis* PB-SRB1	5	63,498	16,429	15,320	11	16	8	0	0	0
7	*Thiohalocapsa* PB-PSB1	37	8,348,936	3,497,020	450,066	231	507	16	99	6	42
1 + 6	*Desulfofustis* PB-SRB1	39	4,114,077	605,607	231,618	46	74	8	99	0	0
4 + 5	*Rhodobacteraceae*	51	4,776,701	784,839	219,055	27	46	7	95	2	77

Metagenome-assembled genomes (MAGs) with completeness >90% are represented by methylation group 7, groups 1 + 6, and groups 4 + 5.

^a^Completeness was assessed by presence of lineage-specific single copy marker genes.

^b^Contamination was assessed by the presence of single copy marker genes in >1 copy.

^c^Strain heterogeneity is the proportion of observed multicopy marker gene sets sharing >97% amino acid identity. For example, nine marker genes in the Rhodobacteraceae MAG (clusters 4 + 5, bottom line in table) were found in duplicate copies. In seven of these nine genes though, their duplicate copies shared >97% aa identity, indicating these “contaminants” derived from highly similar strains or incomplete assemblies, rather than inclusion of distant organisms due to binning errors (e.g., 77% strain het. = 7/9).

### Binning and circular assembly of the *Thiohalocapsa* sp. PB-PSB1 genome (Group 7)

Group 7, the largest of the methylation groups at 8.3 Mb, represented a 99% complete MAG for *Thiohalocapsa* sp. PB-PSB1, the most abundant organism in the consortia (Table 1) [29, 30]. This group contained long contigs (N50 450 kb, max 3.5 Mb), unlike corresponding Illumina MAGs, which were far more fragmented (N50 ~40 kb, max 160 kb). Contigs in this group larger than 100 kb (*n* = 15) had an average coverage of 489×, while the smaller contigs (<25 kb, *n* = 22) were lower coverage with an average of 57× (Supplementary Data 3).

Of the 37 contigs in this group, 31 were clearly identified by sequence similarity as *Thiohalocapsa* sp. PB-PSB1 (Fig. 3A and Supplementary Data 3). Six contigs did not have clear taxonomic assignments but grouped most closely with other PB-PSB1 contigs based on sequence composition (Fig. 3B). Five of these taxonomically unidentified contigs also shared strong assembly graph connectivity with other PB-PSB1 contigs (Supplementary Data 4). Contamination for this group, estimated based on the percentage of single copy marker gene sets present in multicopy, was predicted to be 6.6%. However, 42% of these multicopy marker genes (14 in total) shared ≥99% amino acid identity (aai) between copies, and no multicopy genes revealed hits to distantly related taxa. These multicopy genes, therefore, likely do not indicate contamination, but rather strain-level variation, incomplete assembly, or recent duplications.

Within methylation group 7, a subset of seven, smaller contigs (7a, length <25 kb, coverage 46 ± 7×) shared an unusual methylation profile relative to other contigs. While the contigs in clade 7a encode the sequence of the characteristic PB-PSB1 motifs m8, m13, m14, m18, and m19, these motifs were rarely methylated. By contrast, these motifs were almost universally methylated when they occurred on the other contigs in methylation group 7, even on contigs with less than 40× coverage.

Reassembly of group 7 sequence data produced a circular assembly graph formed by nine backbone contigs (Supplementary Data 4). In addition, there were 51 small contigs forming “bubbles” or spurs connected to this main assembly graph (length <25 kb, 543 kb total), and four “singleton” contigs unconnected to the circular assembly graph (length <22 kb, 57 kb total). With manual curation, the genome was closed to produce a single circular contig of 7.95 Mb which represents the finished genome of *Thiohalocapsa* sp. PB-PSB1 (CP050890). Marker gene analysis of this assembly identified only eight duplicated markers out of a set of 581 (1.4%), with no marker duplicates sharing ≥97% aai (Supplementary Information). This finished genome contains 606 IS elements that comprise 9.4% of the total genome sequence (Table 2). These IS elements were both diverse, belonging to 17 phylogenetically distinct families, and highly repetitive as demonstrated by a 1.5 kb IS154 transposon found in 44 identical copies distributed throughout the genome. Insertion or deletion of IS elements contributed to the structural variants, observed as bubbles in the assembly graph (Supplementary Fig. 5).

**Table 2:** The number insertion-sequence (IS) elements from different families in the finished, circularized genome of *Thiohalocapsa* sp. PB-PSB1 compared to other publicly available *Thiohalocapsa* genomes: *T*. ML1 (NZ_JABX01000001), *T. halophila* (NZ_NRRV01000001), and *T. marina* (NZ_VWXX01000010).

Family^a^	*T*. PB-PSB1	*T*. ML1	*T. halophila*	*T. marina*
IS4	190	10	4	1
IS91	120	10	3	5
ISL3	54	0	0	0
IS5	50	0	1	0
ISAS1	35	3	4	2
IS630	29	3	10	5
ISAZO13	18	1	1	0
IS110	16	1	2	3
IS66	16	0	4	4
IS21	15	4	2	2
IS1634	15	4	0	0
IS200/IS605	14	0	1	1
IS1182	10	0	0	0
ISKRA4	9	0	1	0
IS701	7	0	0	0
IS3	4	2	1	2
ISNCY	3	11	3	3
IS1380	0	0	3	1
IS256	0	0	1	3
IS481	0	0	0	1
IS1595	0	1	4	0
New	1	2	0	0
Total	606	52	45	33
Genome size (Mb)	7.9	6.3	5.7	4.3
% of genome	9.42	0.99	0.84	0.97

^a^IS elements were classified into phylogenetically distinct families based on the ACLAME database.

### Identifying HGT in *Desulfofustis* sp. PB-SRB1 (Groups 1 and 6)

Methylation groups 1 and 6 comprised the complete (99%) and uncontaminated (<0.5%) genome of *Desulfofustis* sp. PB-SRB1. Sequence similarity and composition both confirmed that these two methylation groups contain contigs originating from a common species, highly similar to prior data from *Desulfofustis* sp. PB-SRB1 (Fig. 3B) [29]. Group 1 shared some common methylated motifs with PB-PSB1 (e.g., m3 and m10), but also contains other frequently methylated motifs (m20–m27) that were unique to PB-SRB1 (Fig. 2). Though the methylation profiles of contigs in groups 1 and 6 differ from one another (Fig. 3A), they shared several key similarities, namely, the methylation of motifs m20, m3 and m21 and absence of methylation on other motifs (Fig. 2). These two groups differed significantly in coverage: group 1 contained the majority of the genome at ~50× coverage, while group 6 contained only 63 kb at ~11× coverage (Table 1). Many group 6 contigs shared sequence similarity with larger, higher coverage portions of the assembly in group 1 and may represent structural variants (some had transposon deletions or sequence rearrangements relative to their parent contigs).

**Fig. 4:**
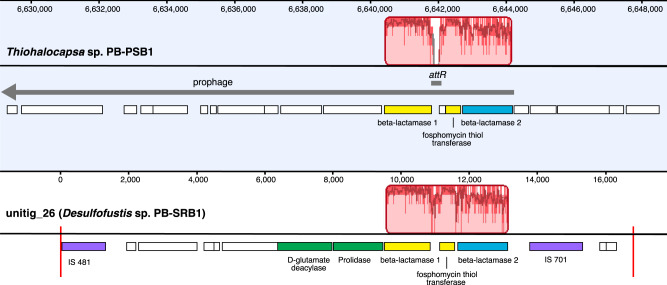
Genome alignment shows evidence for the horizontal transfer of antibiotic resistance genes between the bacterial symbionts. Shown are the aligned genomes of *Thiohalocapsa* sp. PB-PSB1 (top, finished genome) and *Desulfofustis* sp. PB-SRB1 (bottom, unitig_26). Highlighted in red is the homologous region identified by whole genome alignment, where bar height represents the degree of conservation. Highlighted in yellow are the highly conserved genes: beta-lactamase 1 (88% nucleotide identity; 97% aa similarity) and a fosphomycin resistance thiol transferase (91% nt id; 97% aa similarity). Beta-lactamase 2 (in blue), which contained an N-terminal twin arginine leader peptide, was less closely related (74% nt id; 88% aa sim). On unitig_26, this region was flanked by transposons (purple) found on several other contigs in the *Desulfofustis* sp. PB-SRB1 assembly. In the *Thiohalocapsa* sp. PB-PSB1 genome, this region falls within a 29 kb prophage (gray arrow). The *attR* insertion site (black line) for the prophage is not conserved in the unitig_26 sequence, as evidenced by the dip in sequence similarity in this region.

One 10 kb contig, unitig_146, clustered with group 1 in both hierarchical and t-SNE methylation clustering but was most similar to *Thiohalocapsa* sp. PB-PSB1 contigs in sequence composition (Fig. 3). Given the conflicting evidence, we further investigated this contig to determine whether this represented HGT or a binning error. We manually inspected read alignments and the assembly graph for this contig in the reassembled PB-SRB1 genome and found no evidence of misassemblies. This contig encodes two class C beta-lactamase genes alongside a D-glutamate deacylase and prolidase, functions that suggest that this gene cassette enables both the opening of beta-lactam rings and decarboxylatation to their constituent D-amino acids (Fig. 4). Flanking these genes were two transposons (IS481 and IS701) most closely related to homologs from the *Desulfobacterales* and found in multiple copies on the other contigs in the PB-SRB1 genome. Neither of these transposons were found in the closed genome of *Thiohalocapsa* sp. PB-PSB1. This contig was in a complex region of the PB-SRB1 assembly graph, with connectivity to two large contigs containing PB-SRB1 marker genes (>100 kb), and two smaller contigs (<10 kb). Alignment of these contigs and their component reads revealed numerous structural variants in this region (Supplementary Fig. 6). Combined with the distinctive methylated motifs present on this contig, these findings give us confidence in our assignment of this contig as a true portion of the PB-SRB1 genome.

Alignment of this *Desulfofustis* sp. PB-SRB1 contig with the closed *Thiohalocapsa* sp. PB-PSB1 genome revealed sequence similarity only in the 3.7 kb region containing these two beta-lactamase genes (Fig. 4). This region of the PB-PSB1 genome overlaps with a 29 kb prophage, complete with flanking *attL* and *attR* insertion sites. However, this prophage region was not conserved in PB-SRB1.

### Resolving three distinct *Alphaproteobacteria*

The remaining methylation groups 2–5 are composed of contigs from three different *Alphaproteobacteria*. Motif m29 (GANTC) was frequently methylated on nearly all contigs from groups 2–5. Group 3, which was characterized by frequent methylation of motif m28 (RGATCY) in addition to m29, represents a partial and uncontaminated MAG closely related to *Oceanicaulis alexandrii* (Figs. 2 and 3 and Table 1).

### A novel genus in the *Rhodobacteraceae* (Groups 4 and 5)

Binning together methylation groups 4 and 5, we recovered a 4.7 Mb, 95% complete MAG with 2% contamination corresponding to strain heterogeneity (Table 1). This long-read bin shared 99.8% ANI with a 4.2 Mb MAG from our Illumina dataset (PB-A2), estimated to be 97.5% complete with 0.4% contamination. By tree placement and ANI (99%), these MAGs’ closest relative in public databases is UBA10424 (GCA_003500165.1, N50 = 13 kb), an 88% complete MAG extracted from our previous, lower coverage sequencing of this same system in 2010, and proposed to be the sole representative of a novel genus in the *Rhodobacteraceae*.

While these groups were separated in methylation space, they clustered closely together based on sequence composition (Fig. 3). Group 5 contained the majority of the genome (4.5 Mb), while group 4 contained smaller, lower coverage fragments (Table 1). Many of the high GC contigs in these methylation groups could be identified by sequence similarity as belonging to the family *Rhodobacteraceae*. Contigs without clear taxonomic identity could be linked with the other *Rhodobacteraceae* contigs based on their overlap-based assembly graph connectivity (Supplementary Data 3-4). These groups shared methylation of m28 and m29 with group 3 (*Oceanicaulis*) but were distinguished by the methylation of m30 (CANCATC) and m32 (GATGGA).

Group 4 contained three low GC contigs from the *Bacteroidetes* that represent contamination (black arrow Fig. 3). Though these contigs did contain detected modifications (Supplementary Data 1), these methylations either never (unitig_245) or rarely (unitig_260, unitig_174) occurred within one of the 32 characteristic motifs. These data suggest that these contigs clustered with the lowest coverage contigs (cluster 4) in our dataset based on the absence of methylated motifs, rather than any positive signal.

### Linking phage infection with a novel *Micavibrionaceae* species (Group 2)

Group 2 comprises 158 kb of sequence on six contigs, four of which were identified as belonging to the *Micavibrionaceae* by sequence similarity (Table 1). The methylation profile of group 2 contained m29, like the other *Alphaproteobacteria*, but was missing m28. Group 2 was further distinguished by distinctive combination of methylated motifs m25, a 4mC motif (CCAGCG), and m11 (GAGATG). The contigs identified as *Micavibrionaceae* (30× coverage) mapped with high identity to a MAG (PB-A3) binned from our parallel Illumina assembly (84% complete, 0.5% contamination, N50 32 kb). This MAG’s closest relative in public databases is UBA10425 (GCA_003499545.1), an 80% complete genome extracted from our prior, lower coverage sequencing of this same system, and proposed to be the sole representative of a novel genus within the *Micavibrionaceae*.

The remaining two contigs in group 2 were present at significantly higher coverage (70× and 110×) and were identified as putative phage sequences. While these contigs clustered closely with the others based on their methylation profiles (Fig. 3A), they had markedly different sequence composition relative to the other *Micavibrionaceae* contigs (Fig. 3B). The first of these, unitig_102, was an outlier at 110× coverage, which was the highest coverage contig in this dataset that was not from *Thiohalocapsa* sp. PB-PSB1. This 75 kb contig is predicted to encode a complete *Siphoviridae* dsDNA phage genome, with both structural and DNA replication genes (Supplementary Fig. 7A). Ten of these coding sequences shared high percent identity (32–66% aai) with a cultured temperate phage, phiJI001, known to infect an alphaproteobacterial isolate from the genus *Labrenzia* (Supplementary Fig. 7B). Searches of this *Siphoviridae* contig against our Illumina-based MAGs found high percentage identity matches to several contigs binned to *Micavibrionaceae* PB-A3 (>99% nucleotide identity). These contigs were linked to the PB-A3 bin based on paired-end read connectivity, but not by our sequence composition or coverage-based analyses. Unitig_102 could be circularized (with manual trimming), a common characteristic of *Siphoviridae* genomes; however, the PacBio data and Illumina paired-end reads both supported scaffolding with a 100 kb contig in the Illumina assembly that contained *Micavibrionaceae* marker genes.

### Methylation-based clustering is more accurate or more complete than automated binning algorithms using tetranucleotide and coverage information

We compared the performance of our methylation-based clustering to two widely used, automated binning algorithms that analyze tetranucleotide and coverage information, MetaBAT2 [31] and MaxBin [32]. MetaBAT2 recovered seven bins ranging from 230 kb to 6.8 Mb in size with very low (<2%) contamination and variable completeness (0–92%), based on lineage-specific marker genes analysis (Supplementary Table 4). Each MetaBAT2 bin contained contigs belonging to only one methylation group. However, MetaBAT2 conservatively left more than 1.7 Mb of sequence unassigned, and consequently, the MetaBAT2 bins were smaller than our methylation groups, recovering between 5% and 84% that sequence data. Even combining across bins, the *Desulfofustis* sp. PB-SRB1 and *Thiohalocapsa* sp. PB-PSB1 MAGs from MetaBAT2 recovered only 95% and 96% (respectively) of the genome sequence binned using methylation profiles (Supplementary Table 4). Notably, the putative HGT contig (unitig_146) remained unassigned, as were group 7a contigs (*Thiohalocapsa*), and all contigs from *Micavibrionaceae* and their phage (Supplementary Data 3).

MaxBin created three larger bins between 4.5 and 8.0 Mb with completeness estimated between 96% and 99% (Supplementary Table 5). However, two of these bins contained substantial contamination (5% and 26%) based on marker gene redundancy. The highest quality bin, corresponding to *Thiohalocapsa* sp. PB-PSB1, recovered 97% of the sequence from methylation group 7. Notably, however, MaxBin misassigned all of the *Thiohalocapsa* contigs below 480× (including those from group 7a) by grouping them instead with the *Desulfofustis* sp. PB-SRB1 sequence (Supplementary Data 3). This *Desulfofustis* MAG created by MaxBin, though complete, contained further contamination from all three *Alphaproteobacteria*. The final bin (with 26% estimated contamination) was a mixture of all three alphaproteobacterial MAGs that we were able to resolve separately using their methylation profiles (Supplementary Table 5).

### Novel restriction-modification (RM) systems and orphan methyltransferases explain the diversity of methylation patterns in the metagenome

**Fig. 5:**
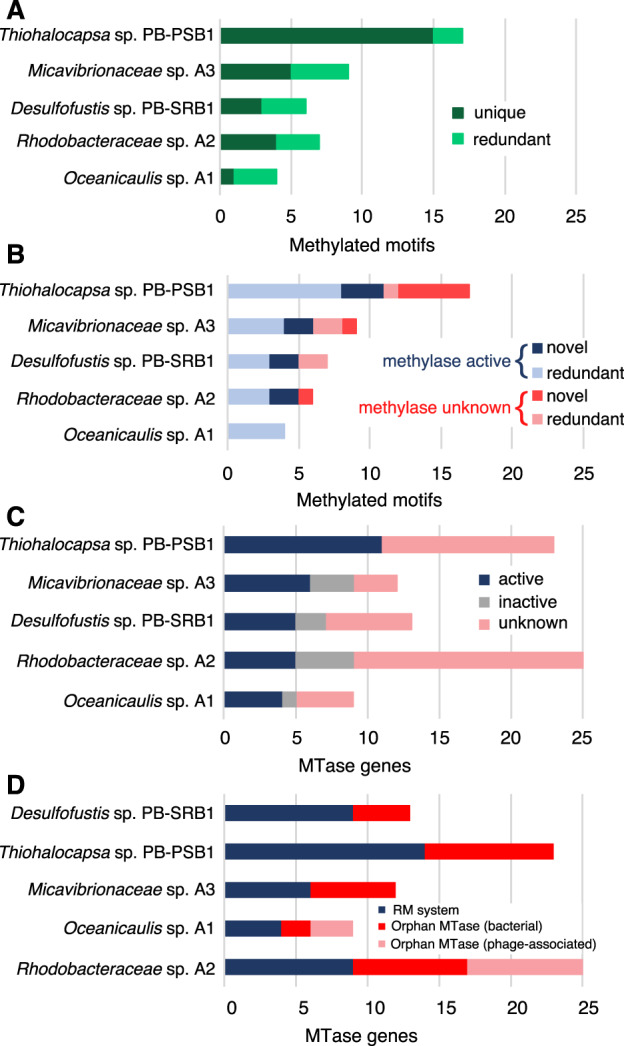
Novelty and distribution of methylated motifs and MTase genes. **A** Analysis of each metagenome-assembled genome (MAG) demonstrates that while some methylated motifs were observed amongst several consortia members (redundant, light green), each MAG contained methylated motifs unique to that species in the dataset (unique, dark green). **B** Most methylated motifs in each MAG could be linked with a predicted source MTase (light blue, navy), though each genome (except for *Oceanicaulis)* had some motifs for which the source MTase remains unknown (pink, red). All genomes except for *Oceanicaulis* sp. A1 contained novel motifs yet to be documented in REBASE (red, navy), while others were redundant with known RM recognition sequences (light blue, pink). **C** Each organism contained numerous MTase genes, which were classified as “active” (navy blue) when MTase’s predicted recognition sequence was methylated, “inactive” (gray) where the predicted recognition sequence was not frequently methylated, or “unknown” (pink) if the recognition sequence could not be predicted bioinformatically. **D** Approximately half of all MTase genes in each genome were observed alone, without an adjacent restriction enzyme (“orphan” MTases). In some genomes, orphan MTase were associated with phage genes or contigs (pink), while others were not (red).

To further investigate the patterns of DNA methylation in the consortia, we analyzed each MAG individually that detects methylated motifs with greater sensitivity. For the incomplete genomes (e.g., the *Alphaproteobacteria*), we also analyzed their corresponding Illumina-assembled MAGs as validation. The genomes each contained from 4 to 17 different methylated motifs, and every genome had at least one methylated motif unique to that organism in the consortia (Fig. 5A). This analysis recovered 30 of the 32 motifs from our initial prediction, and also discovered 12 additional methylated motifs (Supplementary Data 5). There is substantial novelty in these genome modifications: 40% of these methylated motifs have never been reported in genome-wide methylation studies or databases of RM recognition sites (*n* = 17; red and navy bars in Fig. 5B) [33].

We investigated the source of these methylation patterns by annotating the MTase and restriction enzyme genes in each genome. We found between 9 and 24 different MTase genes in each genome, and for ~50% of these genes, we could bioinformatically predict their recognition sequences, many of which matched methylated motifs in the genomes (Fig. 5 and Supplementary Data 5). Every genome, except for *Oceanicaulis*, encoded 2–3 novel RM systems that we predict recognize and methylate (or cut) novel sequence motifs (navy blue bars, Fig. 5B). Approximately half of the MTase genes from these genomes (40/82) were “orphan” Type II MTases, found without a cognate restriction enzyme (Fig. 5D). Though many of these orphan MTases were either inactive or unassigned, we observed widespread GANTC modifications (motif m29 in Fig. 2) associated with ﻿cell cycle–regulated methyltransferase (CcrM) homologs in all three of our alphaproteobacterial genomes (Supplementary Fig. 8 and Supplementary Data 5). CcrM family MTases, which are highly conserved orphan MTases amongst *Alphaproteobacteria*, are known to methylate GANTC and function as key regulators of the cell cycle [34, 35].

Several orphan MTases in the *Oceanicaulis* and *Rhodobacteraceae* MAGs were found to be encoded on putative phage or prophage contigs (Fig. 5D). Though most phage MTases were orphans, one 60 kb phage contig in the *Rhodobacteraceae* MAG encoded 3 Type II orphan MTases, as well as a complete Type I RM system (Supplementary Data 5). These sequences were quite divergent from known MTases, and as such their recognition sites could rarely be predicted, with the exception of GATC phage MTases which would likely confer protection against the hosts’ RGATCY cleaving restriction enzymes.

**Fig. 6:**
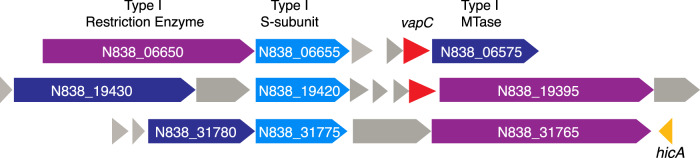
Type I RM systems in *Thiohalocapsa* sp. PB-PSB1 co-occur with the RNase toxins from the *vapC* (red) or *hicA* (orange) families. These loci did not include the well-described *vapB* or *hicB* family antitoxins, though these were encoded elsewhere in the genome.

Examining the RM systems in the *Thiohalocapsa* sp. PB-PSB1 genome, we discovered that RM genes frequently co-occurred with putative RNase or RNA interferase toxin genes from the *vapC* or *hicA* family. Six of the 23 MTases in this genome were immediately flanked by these *vapC* or *hicA* toxins. Five of these cases encoded complete RM systems—including three out of the four complete Type I operons in the genome (Fig. 6 and Supplementary Data 5). These loci encoded only the toxin gene without their described antitoxin; however, *vapB* and *hicB* family antitoxins were found elsewhere in the genome.

## Discussion

Examining metagenomic methylation patterns, we binned and assembled complex bacterial genomes from a microbial consortium with substantial strain variation. Such methylation-based binning has been tested using cultured mock communities and the gut microbiome [23, 36, 37]; however, this approach has yet to be validated in other ecosystems. Though we use a different workflow in identifying methylated motifs, we similarly found that methylation patterns faithfully distinguish contigs from distinct species. We identified the host for a complete phage genome based on their similar patterns of DNA methylation, the first application of this novel approach for linking phages with their hosts. Compared to automated algorithms based on tetranucleotide frequency and coverage, methylation profiling was more sensitive than MetaBAT2 and more accurate than MaxBin.

With our approach, we finished the largest and most complex circular bacterial genome yet recovered from a metagenome. Though closing genomes is now routine with bacterial and archaeal isolates, circularized metagenome-assembled genomes (cMAGs) remain rare and tend to be both clonal and small [13], though they are becoming increasingly accessible with long-read sequencing [38]. At 7.9 Mb, the circularized genome of *Thiohalocapsa* sp. PB-PSB1 is the largest finished genome ever reconstructed from a metagenomic sample, exceeding next largest, a long-read pseudomonad cMAG, by nearly 1.5 Mb [39]. This is also the largest in this family (*Chromatiales*), though some *Chromatiales* have genomes in excess of 6 Mb.

Previous short-read metagenomes recovered complete but highly fragmented genomes for the most abundant species in the consortium, *Thiohalocapsa* sp. PB-PSB1 and *Desulfofustis* sp. PB-SRB1 [29], which suggested strain complexity or intragenomic repeats. Indeed, the finished *Thiohalocapsa* sp. PB-PSB1 genome is highly repetitive: it harbors amongst the highest number of transposons ever reported in a bacterial or archaeal genome [40, 41]. With 9.4% of its genome comprising transposon sequence, *Thiohalocapsa* sp. PB-PSB1 has an unusual genome structure for free-living bacteria, though not unprecedented among aggregate- and bloom-forming phototrophs [42, 43]. Transposable element expansions of this scale are more commonly observed in host-restricted bacteria, such as pathogens and endosymbionts [44]. Though transposase activity is typically thought to be kept low to avoid genome degradation, a relative of *Thiohalocapsa* sp. PB-PSB1, the gutless worm endosymbiont Cand. *Thiosymbion algarvensis* has abundant, diverse, and highly active transposases [45, 46]. Repetitive mobile elements are not only vehicles for transposition and HGT, but also frequently flank hotspots of homologous recombination in bacterial genomes [47, 48]. The transposon abundance in *Thiohalocapsa* sp. PB-PSB1, thus, indicates substantial potential for recombination and genome plasticity.

Strain-level structural variants of transposons (e.g., deletions, inversions) were evident in both the PB-PSB1 assembly graph and in mapped reads spanning transposon regions in the finished genome. In the hierarchical clustering of contigs by methylation profile, we observed a clade of small contigs from PB-PSB1 where several distinctive sequence motifs remained unmethylated (Fig. 2, group 7a). These sequences were structural variants of the finished, circular genome and contained transposons that we found, in different sequence contexts, elsewhere in the finished genome. Considered together, this evidence suggests that sequences in group 7a originate from a distinct strain (or strains), distinguished from the finished PB-PSB1 cMAG by genome rearrangements near transposons and missing or inactive MTases. While these missing methylations could be an artifact of low coverage, we find this interpretation unlikely as these motifs were frequently methylated on many lower coverage contigs in PB-PSB1 and coverage as low as 15× can reliably detect Type I motif methylations [24].

The *Desulfofustis* sp. PB-SRB1 genome was complete but remained draft quality (*n* = 72, N50 385 kb, max 930 kb), due to strain-level structural variants and lower coverage. Methylation profiling provided key information allowing us to link an island of horizontally transferred antibiotic resistance genes to the *Desulfofustis* sp. PB-SRB1 genome. This small contig was either unbinned or erroneously grouped with the *Thiohalocapsa* sp. PB-PSB1 genome by composition and coverage-based algorithms; however, we were able to correctly identify it as belonging to *Desulfofustis* sp. PB-SRB1 based on its distinctive methylation profile.

The patterns of methylation in the pink berry MAGs are highly novel and offer a window into unexplored microbial DNA methylation systems: 40% of methylated motifs we found have no matches in restriction enzyme databases [33]. Systematically annotating the MTase genes in each genome, we discovered 7 RM systems that we predict recognize some of these novel methylated motifs. The majority of these novel MTases were Type I systems recognizing asymmetric target sites with the nonspecific spacer of 4–8 bp (typically 6 bp), characteristic of Type I RM systems [25]. *Thiohalocapsa* sp. PB-PSB1 remains a rich target for discovery, with a dozen uncharacterized MTases and five novel methylated motifs without a predicted MTase (Fig. 5B, C). Clearly, further experimental characterization of these MTases and restriction enzymes is warranted and could yield enzymes of biotechnological utility [49], a finding echoed by a recent study investigating the methylation patterns and experimentally validating novel RM systems from aquatic bacteria [50].

In addition to RM systems, we also observed Type II orphan MTases in all of these MAGs. While we cannot exclude the possibility that cognate restriction enzymes for some orphan MTases went undiscovered due to sequence divergence or incomplete assemblies, our observations are consistent with findings that active orphan Type II MTases are diverse and widespread amongst cultured bacteria and archaea [24]. In two genomes (*Oceanicaulis* sp. A1 and *Rhodobacteraceae* sp. A3), some orphan MTases were associated with phage contigs. Such phage-encoded MTases have been reported in ~20% of all phage genomes, and, amongst those that have been studied experimentally, they help evade the host’s restriction enzymes or regulate the lytic/lysogenic lifecycle [26, 51]. Bacterial orphan MTases, previously relegated solely to housekeeping roles like facilitating DNA mismatch repair, have more recently been found to play key regulatory roles in processes including cell cycle control, virulence, adhesion, and biofilm formation [26]. In all the alphaproteobacterial MAGs, we found the genes and corresponding GANTC methylation for ccrM-family MTases, a highly conserved orphan MTase that regulates the cell cycle in cultured *Alphaproteobacteria* [34, 35].

Studies of GATC-modifying Dam orphan MTase in cultured gammaproteobacterial pathogens suggest several intriguing hypotheses about the bacteria in the pink berry consortia. Protein binding at GATC sites can block Dam methylation. In some promoter regions, this phenomenon results in heritable, bistable methylation patterns that produce phenotypic variants that are genetically identical [reviewed by 26]. Such epigenetic regulation by phase variable MTases opens up new possibilities for multicellular bacterial populations, like those in the pink berry consortia, including the division of labor between these “differentiated” cells or evolutionary bet-hedging between current conditions and future challenges [26].

In *Escherichia coli*, Dam-dependent methylation of GATC sites in transposase promoters has also been demonstrated to repress the activity of transposons from the IS4 and IS5 families (IS10, IS50, IS903) and limit the expression of transposases to the period immediately after DNA replication [52–54]. Given that IS4 and IS5-family elements together comprise almost 3% of *Thiohalocapsa* sp. PB-PSB1’s genome (Table 2), a similar role for orphan MTases would give these bacteria physiologically and evolutionarily critical control over transposition.

In the PB-PSB1 genome, we discovered RNA-targeting *vapC* and *hicA* toxin genes immediately adjacent to RM systems, a co-occurrence that has not previously been reported (Fig. 6). We propose that these VapC and HicA homologs play a role in phage defense or programmed cell death, analogous to the PrrC-Ecoppr1 abortive infection system in *E. coli* [55]. Though these systems do not share sequence homology, the functional parallels are notable. The PrrC abortive infection system includes an anticodon tRNA nuclease which initiates programmed cell death, should the Type I restriction enzyme defense fail against phage infection (Supplementary Fig. 9) [reviewed by ref. [56]]. Bioinformatic analyses predict widespread occurrence of such complexes between HEPN-domain RNAses (like PrrC) and RM systems in *Bacteria*, though none have been experimentally characterized beyond *E. coli* [57]. Our preliminary investigations revealed that *vapC* or *hicA* homologs also co-occurred with RM genes in other bacterial genomes (Supplementary Data 6). Together, these discoveries suggest that diverse RNA-acting toxins may be more widely integrated as a “fail-safe” defense with restriction enzymes than was previously appreciated.

From an evolutionary perspective, abortive infection provides a valuable strategy for multicellular, biofilm-dwelling bacteria like *Thiohalocapsa* sp. PB-PSB1, where a single infected cell poses a grave risk of infection to its susceptible kin. We observed that toxin homologs colocalized with RM systems were often from bacteria known to have multicellular life forms (e.g., filamentous cyanobacteria like *Microcystis aeruginosa* or rosette-formers like *Nevskia ramosa* [58], Supplementary Data 6). Diverse RNase-RM complexes may also play roles beyond phage defense and apoptosis: *vapBC* and *hicAB* have been previously characterized as bacteriostatic Type II toxin-antitoxin systems that regulate translation and growth rates and play diverse physiological roles, from stress response to host-microbe interactions [59]. Studies further investigating co-occurrence of these systems in sequenced genomes and the interactions of these target proteins present a promising avenue for future inquiry of these putative complexes and their role in bacterial ecophysiology.

DNA methylation patterns provide a novel and informative addition to the suite of genomic signatures we analyze to bin and refine metagenomic sequence data. Simulations based on databases of cultured bacteria suggest that, even in more complex communities with co-existing strains (~200 genomes), more than 80% of taxa will have unique genome-wide methylation patterns [23]. Both PacBio and Oxford Nanopore are capable of DNA methylation detection with at least 20× contig coverage; with sequencing flow cells now yielding 20–50 Gb, such an approach is feasible for more diverse communities. Automated binning algorithms based on tetranucleotide frequency and coverage were unable to correctly assign contigs enabling key discoveries including HGT, strain-level structural variants, and phage. While including more samples for differential coverage analysis would marginally improve these algorithms’ performance, such an approach still cannot improve binning of those “flexible” genomic regions that vary between samples (and are thus uncorrelated with other portions of the genome). Resolving these complex features in bacterial genomes opens exciting frontiers for investigations of microbial consortia and provides a lens that allows us to examine how ecological interactions—from symbioses to predation—shape bacterial evolution.

## Methods

### Sampling and library preparation

Pink berry aggregates were sampled in July 2011 from Little Sippewissett Salt Marsh (Falmouth, MA, USA), as described previously, and DNA was extracted using a modified phenol chloroform protocol (Supplementary Methods). We created three distinct samples from which DNA was extracted: a very large aggregate ~9 mm in diameter (berry9), a pool of 13 aggregates 2–3 mm in diameter (s01), and a pool of 10 aggregates of similar size (s02). Transposase-based Nextera XT libraries were constructed for samples berry9 and s02 (Illumina, San Diego, CA). Sample berry9 was sequenced via MiSeq (1 Gb of 250 bp paired-end reads), while sample s02 was sequenced with both HiSeq (150PE) and MiSeq (250PE) (Illumina, Supplementary Table 1).

SMRTbell libraries for PacBio sequencing were constructed from 900 ng of berry9 DNA and ~1 microgram of s01 DNA. Sample s01 was size selected by BluePippin, while berry9 was selected with Ampure beads. In total, 42 SMRT cells were sequenced using PacBio RSII from these two libraries using a combination of P4C2 and P5C3 chemistries (25 cells from the berry9 Ampure library and 17 from the s01 BluePippin library). While BluePippin size-selection increased the proportion of reads greater than 8 kb, that library’s sequence yield was poor compared to the more robust Ampure bead library (Supplementary Table 1). The PacBio data were pooled for further processing and are overwhelmingly represented by the sequence data from the berry9 sample (92% of filtered subread base pairs).

### Metagenomic assembly

HiSeq (100PE) and MiSeq (250PE) reads from sample s02 were trimmed and filtered with sga (preprocess -q 20 -f 20 -m 59 --pe-mode=1; [60]), adapter filtered with TagDust [61], and assembled with idba_ud (maxk = 250; v 1.0.9) [62]. This assembly was binned and curated as described previously [29]. Binned sequence was reassembled and the MAGs were quality assessed with CheckM [63].

PacBio sequence data were error corrected using SMRT Analysis 2.2, yielding 474 Mb of error corrected reads. Error corrected reads longer than 7 kb were assembled with the HGAP assembler (v. 3.3) using a reduced genome size parameter (genomeSize = 5,000,000) to increase tolerance of uneven coverage and an increased overlap error rate parameter (ovlErrorRate = 0.10) and overlap length (ovlMinLen = 60) to encourage contig merging. The topology of the assembly graph from PacBio data (based on the Celera Assembler’s “best.edges”) was visualized in Gephi [64] to determine the overlap-based connectivity between fragmented contigs. This connectivity was used as an additional metric for binning validation, analogous to an approach proposed and validated for short-read assemblies [65]. Metagenomic contigs were quality checked and taxonomically identified as described further in the Supplementary Methods.

### Methylation analysis and metagenomic binning

Methylated bases and their associated motifs were detected on all assembled contigs using the SMRT Analysis v. 2.2 module RS_Modification_and_Motif_Analysis.1 with an in silico control model (modification quality value >20). To identify the set of 32 unique motifs used for our clustering analysis (e.g., Fig. 2), we ran the above module individually on each contig longer than 450 kb (*n* = 8) and verified that these large contigs were reasonable, rough approximations of the abundant community members (using plots of contigs’ average GC vs. coverage; Supplementary Data 3). Large contigs were a convenient target for motif discovery because each contig contained (1) sequence derived from a single species (assuming it was not chimeric), (2) many methylation events which provide better statistical support for a motif, and (3) a diversity of sequence contexts to maximize the potential for recovering different motifs. We selected the length threshold for the contigs to minimize the number of individual contigs analyzed, while maximizing the average GC and coverage diversity in this set.

For this set of 32 motifs, we computed the percentage of methylated motifs out of the total instances of that motif on each contig. The vector of percent methylations for all characteristic motifs represents the contig’s methylation profile. Contigs’ methylation profiles were visualized as a hierarchically clustered heatmap with clusterMaker2 [66] in Cytoscape v. 3.5.1 using average pairwise linkage based on the Euclidean distance between square root transformed methylation profiles. Seven groups of contigs were manually defined from this heatmap visualization (Fig. 2), guided by both the structure of the hierarchical clustering dendrogram and the visual similarities in the heatmap. t-SNE of contigs based on either methylation profiles or tetranucleotide frequencies was performed with the Rtsne package [67] and visualized in R with ggplot2 [68]. To compare the t-SNE clustering with the hierarchical clustering visualization, data ellipses were calculated using ggplot2’s stat_ellipse, based on methylation groups defined above (with the assumption that the population is a multivariate t-distribution).

Reads were recruited back to each bin based on these methylation groups (using blasr in SMRT Analysis 2.2) and were reassembled with HGAP to produce the MAGs. The PB-PSB1 MAG was circularized by manual trimming and curation using Geneious (v R11). MAGs were polished with pilon [69] using both Illumina and PacBio data, and corrections were manually verified for short-read mapping errors. Comparative binning based on sequence composition and coverage was conducted using MetaBAT2 [31] and MaxBin [32]. Contigs and whole genomes were aligned and visualized using progressiveMauve [70]. MAGs were taxonomically identified using GTDB-tk [71]; further information on bin quality assessment is described in detail in the Supplementary Methods.

The methylated motifs in each MAG were predicted independently using SMRT Analysis (v. 2.2). For incomplete genomes (e.g., alphaproteobacterial MAGs), both PacBio- and Illumina-assembled versions of the MAG were used as the reference genome used to recruit the PacBio reads for methylation analysis. MTase and restriction enzyme annotation and motif matching was accomplished by comparison of the genome sequences and methylated motifs with the Restriction Enzyme Database (REBASE) [33], as previously described [24]. Further functional annotation of the MAGs is described in detail in the Supplementary Methods.

## Supplementary information

Supplementary information, figures and tables

Supplementary Data 4

## Data Availability

All sequence data have been deposited in DDBJ/ENA/GenBank under BioProject PRJNA684324. The accession numbers for the Short Read Archive and genome/metagenome data are provided in Supplementary Data 7.
