# Supplementary material for: Metagenomic methylation patterns resolve bacterial genomes of unusual size and structural complexity
Source: ISME J. 2022 Apr 22;16(8):1921–31. doi: 10.1038/s41396-022-01242-7 (PMC9296519; doi:10.1038/s41396-022-01242-7)
Supplement: Supplementary file 1 — Supplementary information, figures and tables [file 41396_2022_1242_MOESM1_ESM.pdf]

Supplemental Methods
Sampling and DNA extraction

"Pink berries" were collected from the sediment-water interface of a shallow marsh pool of the Little Sippewissett Salt Marsh, Falmouth, MA USA (41°34'33.01"N, 70°38'21.24"W) and washed with 0.2 micron filtered marsh water. We created 3 distinct samples from which DNA was extracted: a very large aggregate ~9 mm in diameter (berry9), a pool of 13 aggregates 2-3 mm in diameter (s01), a pool of 10 aggregates of similar size (s02). Berries in each sample were chemically disaggregated by 1 hour incubation at 37 ºC in a 5 M urea solution, pelleted, and washed 3x in 1x PBS in a 1.5 mL tube. High molecular weight DNA was extracted using a phenol chloroform protocol based on that of Dojka, Hugenholtz (1) omitting all bead beating and vigorous mixing. Briefly, samples were resuspended in 500 uL of 2x buffer A (200 mM Tris pH 8, 50 mM EDTA, 200 mM NaCl, 2 mM sodium citrate dihydrate, 10 mM $CaCl_2$ dihydrate) with lysozyme (3 mg/mL), gently homogenized with a pestle and then incubated for 40 minutes at 37 ºC. Samples were gently inverted, and then proteinase K (to 1.2 mg/mL) and sodium dodecyl sulfate (10 uL of 20% w/v, SDS) were added, the tubes gently inverted, and the mixture was incubated for 30 min at 50°C. 500 uL of phenol-chloroform-isoamyl alcohol (phenol:$CHCl_3$:IAA; 24:24:1) and 60 uL of 20% (w/v) SDS were added, placed on a rotating platform for 5 minutes to emulsify, and then samples were spun at 12xkg for 20 minutes at 4 ºC. Supernatant was re-extracted with 1 volume phenol:$CHCl_3$:IAA, and then DNA was precipitated with 1 volume isopropanol and 0.1 volume sodium acetate (3M, pH 5.2) by incubation on ice for 20 minutes followed by 20 minutes spinning at 12xkg at 4 ºC. Pellets were rinsed in 70% cold ethanol and resuspended gently in nuclease free water.

Taxonomic identification, annotation and QC
Open reading frames on every contig were predicted with prokka [2] searched against to NR using BLASTP (v 2.2.25) [3] and taxonomically identified using MEGAN [4]. Taxonomic classification of contigs was based on conserved marker genes where present [5], or else from the consensus of taxonomic assignments from all open reading frames. Where there was no clear taxonomic consensus or if majority of matches were to viral sequences, contigs were labeled "Unclassified" (grey fill, Figure 2). CheckM was used to assess marker gene based completeness and contamination of methylation-based bins [6] and average nucleotide identity (ANI) was calculated according to the OrthoANIu algorithm [7] available at https://www.ezbiocloud.net/tools/ani.

Annotation of MAGs was performed with PGAP [8]. Transposons were identified using ISEScan [9]. Prophage regions and viral contigs were identified using PhiSpy [10] and VirSorter2 [11], respectively. Antibiotic resistance genes were further annotated using the Comprehensive Antibiotic Resistance Database and Resistance Gene Identifier (CARD RGI) [12]. When manually inspecting contigs for misassemblies, we assessed the assembly graph structure and also the mapping of long reads to the contigs to confirm that the contig's consensus sequence was supported by individual read sequence data. Specifically, when examining regions surrounding repetitive regions, we assessed that the repetitive sequence was supported by long reads spanning the repeat element. Read mapping and structural variant detection was performed as described by [13] and visualized using both IGV Genomics Viewer and Ribbon [14, 15].

Supplemental Results

The quality of the manually curated, circularized MAG for Thiohalocapsa PB-PSB1 was further assessed using checkM's lineage specific marker gene analysis. Using their gammaproteobacterial marker set (UID4274) of 581 genes, 557 were found in single copy and 16 were reported in duplicate. Careful analysis of these 16 duplicated markers revealed that, in three cases, two different related markers identified the same set of duplicated genes in the genome, thereby inflating the number of duplicates. For example, the SecA_PP_bind (PF01043.15) and SecA_DEAD (PF07517.9) marker queries both identified genes N838_27410 and N838_09455 as duplicates. This makes the actual percentage of duplicated markers only 2.24%.

For 4 of these remaining 13 duplicated markers, the second hit was a far lower scoring match indicating that it erroneously recruited a distant paralogous gene. This leaves only 8 of 581 markers that may genuinely be duplicated (due to either assembly artifact or biology). Of these, only the RuvA_N (PF01330.16) marker (the Holliday junction branch migration protein RuvA gene) had an amino acid identity suggestive of duplication or strain-level variation (91%). Notably, this marker is also duplicated in other Thiohalocapsa genomes, suggesting a recent duplication in this genus. Duplicates of the other 7 markers all shared amino acid identity of less than 70% (49% + 12, mean+sd). The presence of distantly related copies suggests that these duplications is inconsistent with an assembly marked by chimerism between closely related strains.

To illustrate the potential source of these duplications, we investigate one particular region surrounding the duplicate marker PF01220.14 (DHquinase_II, type II 3-dehydroquinate dehydratase). This marker matched to genes N838_16775 and N838_12800 in the PB-PSB1 genome. The first gene, N838_16775, shares ~80% amino acid identity with homologs from other *Thiohalocapsa* genomes (*T. marina, halophila* and ML-1); while N838_12800 shared 70% aai with a 3-dehydroquinate dehydratase from *Thiothrix nivea*. N838_12800's genomic context is completely distinct from N838_16775, N838_12800's also had high identity to these same species *Thiothrix*; and we note that this region includes elements highly suggestive of recent horizontal gene transfer (GC shift, toxin-anti-toxins, transposases in the vicinity in both). We observed no evidence of misassemblies in this region that would suggest a chimeric assembly: coverage is even throughout this region and individual read mapping strongly supports the assembled sequence. We note that colorless sulfur oxidizers (*Thiothrix*) are abundantly in the natural habitat of the PB-PSB1 presenting a plausible scenario for horizontal gene transfer of this region.

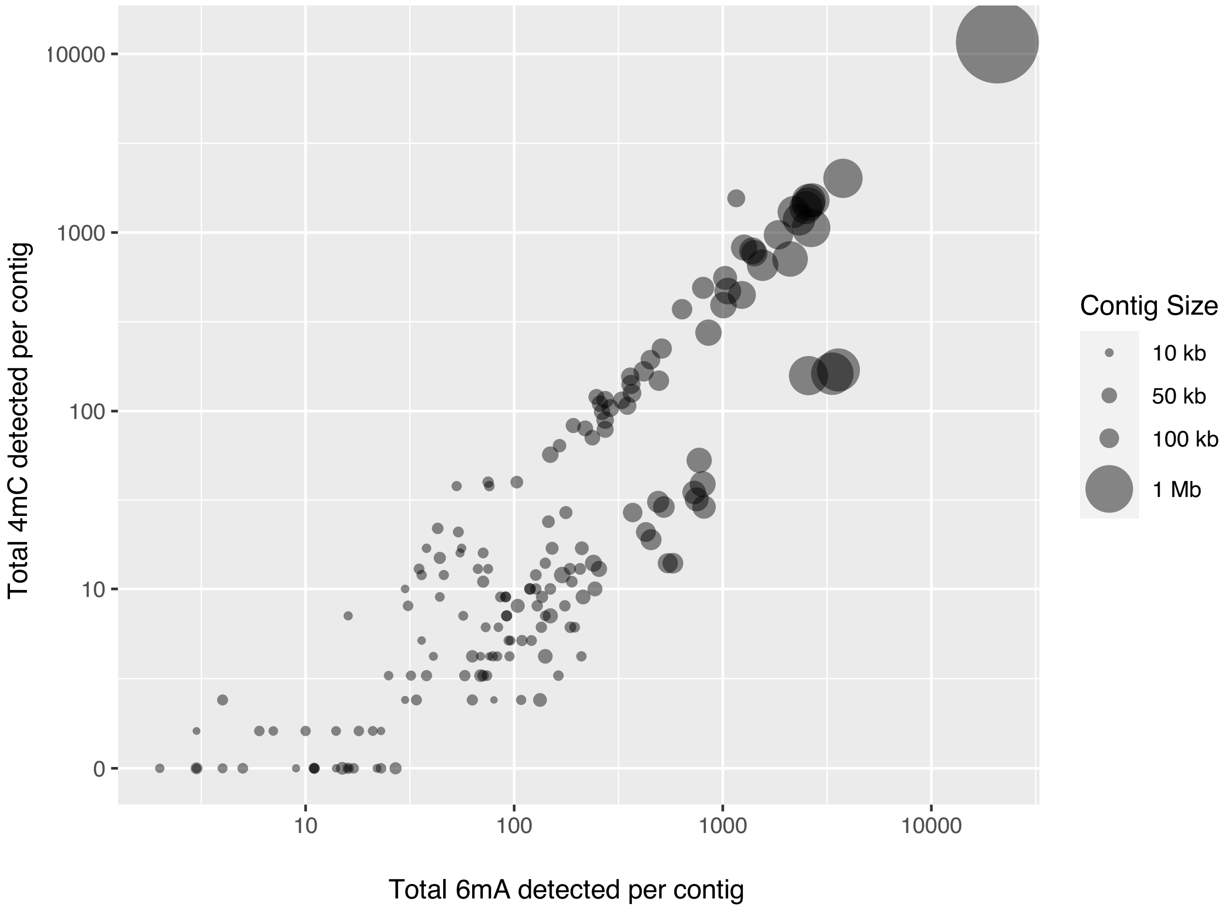

Supplementary Figure 1. The total number of N^6 -methyladenine (6mA) and N^4 -methylcytosine (4mC) detected on each contig in the assembly. Point size is scaled to represent the length of the contig sequence. Methylation detection thresholds were defined as a modification QV ≥ 20, *i.e.* p-value ≤ 0.01).

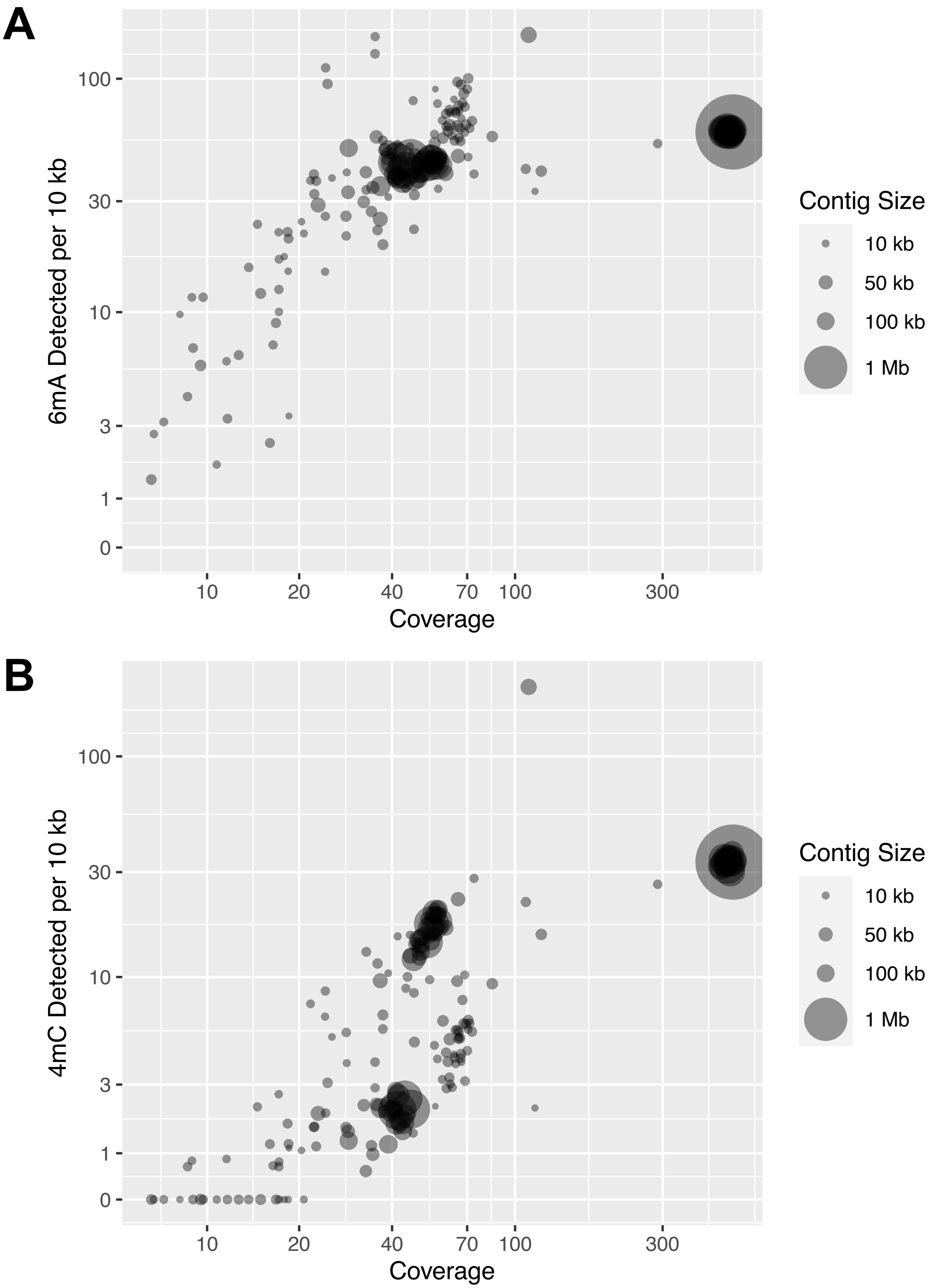

Supplementary Figure 2. The number A) 6mA and (B) 4mC modifications per 10 kb of assembled sequence on each contig. Point size is scaled to represent the length of the contig sequence. Methylation detection thresholds were defined as a modification QV ≥ 20, *i.e.* p-value ≤ 0.01).

Supplementary Figure 3. t-SNE clustering of contigs based on methylation profiles. Equivalent to main text figure 3A but colored by % GC content of the contig. In red are the 3 low coverage, low GC (<45%) contigs within methylation group 4 that were assigned to *Bacteroidetes* (unitig_260, unitig_246) and a putative *Bacteroidetes* prophage (unitig_174).

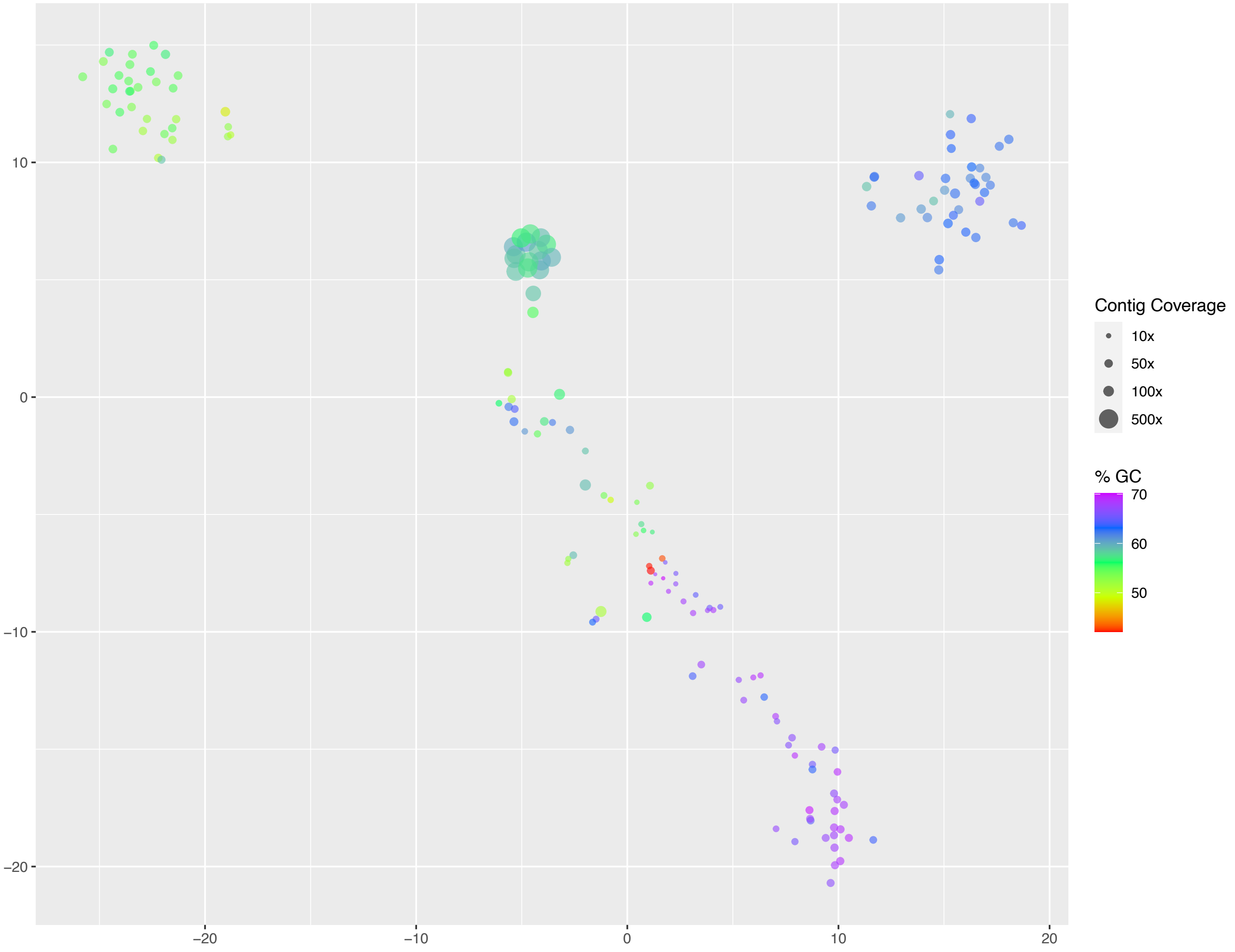

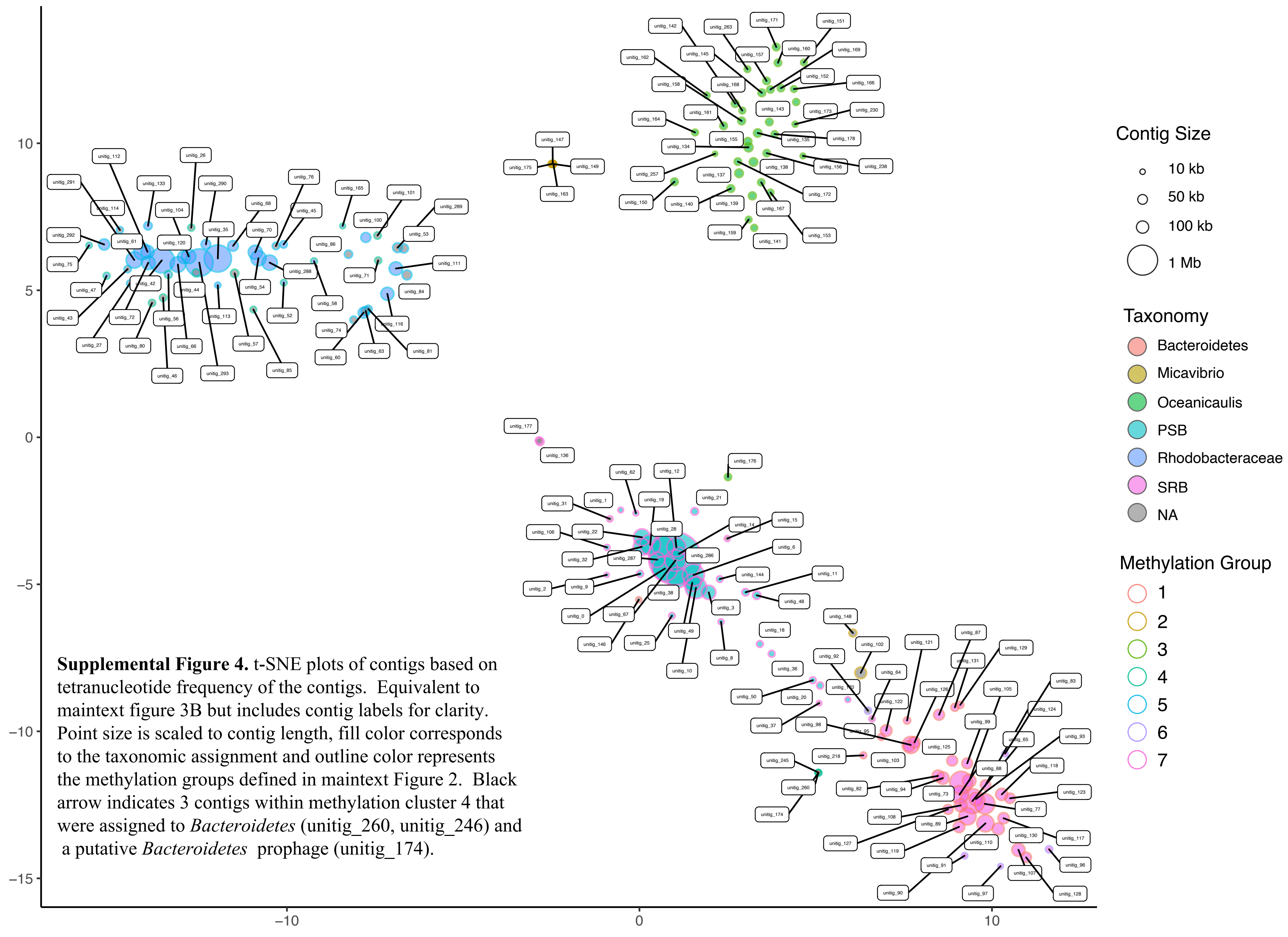

Supplemental Figure 4. t-SNE plots of contigs based on tetranucleotide frequency of the contigs. Equivalent to maintext figure 3B but includes contig labels for clarity. Point size is scaled to contig length, fill color corresponds to the taxonomic assignment and outline color represents the methylation groups defined in maintext Figure 2. Black arrow indicates 3 contigs within methylation cluster 4 that were assigned to *Bacteroidetes* (unitig_260, unitig_246) and a putative *Bacteroidetes* prophage (unitig_174).

Supplemental Figure 5. Two examples are shown of IS-element insertion/deletion which create structural variants visible as “bubbles” in the assembly graph **(A,C)**. The assembly graph **(A,C)** depicts individual reads as the nodes, read overlaps as edges, and the contig assignments as colors and node labels. In the example shown in **A**, the minor variant unitig_44 is missing the IS5 transposon compared to the longer and more abundant variant unitig_3, as seen by the alignment of contigs using progressiveMauve **(B)** and from alignment of an error corrected read visualized as a dot plot using GenomeRibbon **(E).** In the example shown in **C,** the minor variant unitig_23 is missing the ISAS1 transposon as seen from the alignment of contigs **(D)** and an error corrected read **(F)**. The vertical, black rectangle in E and F simply denotes the cursor and can be ignored.

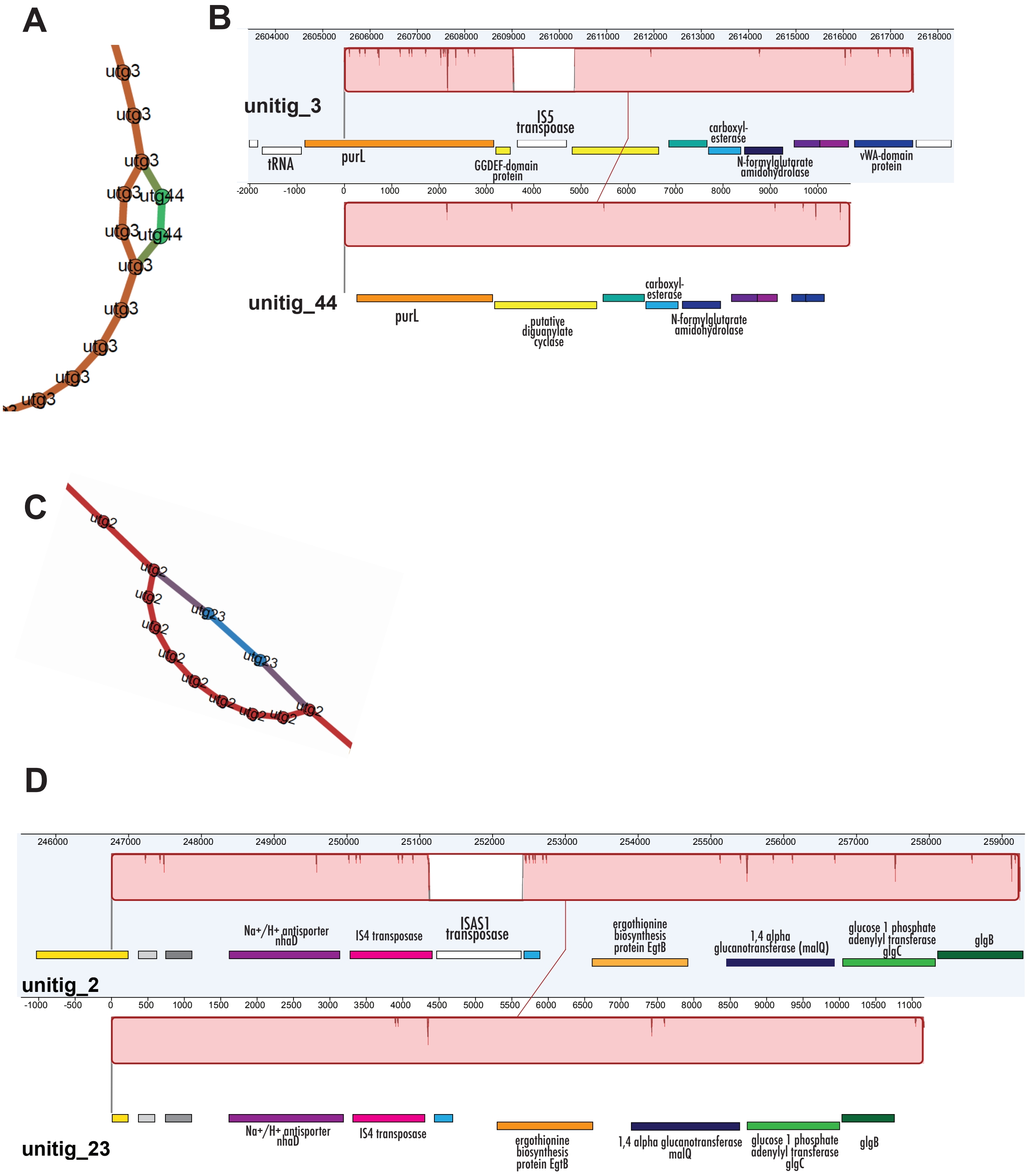

E

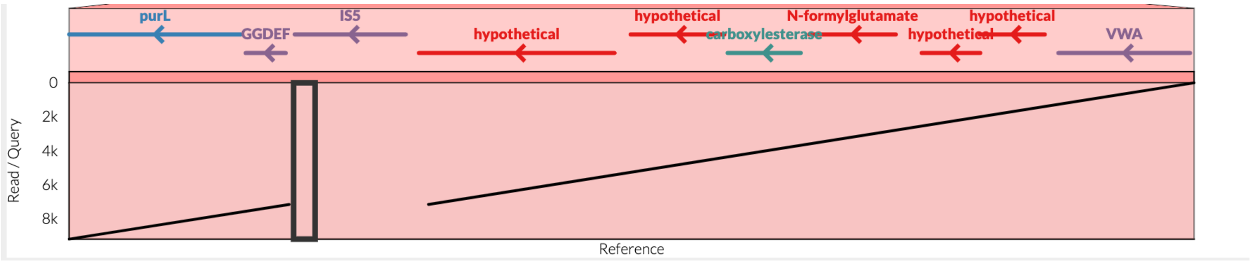

F

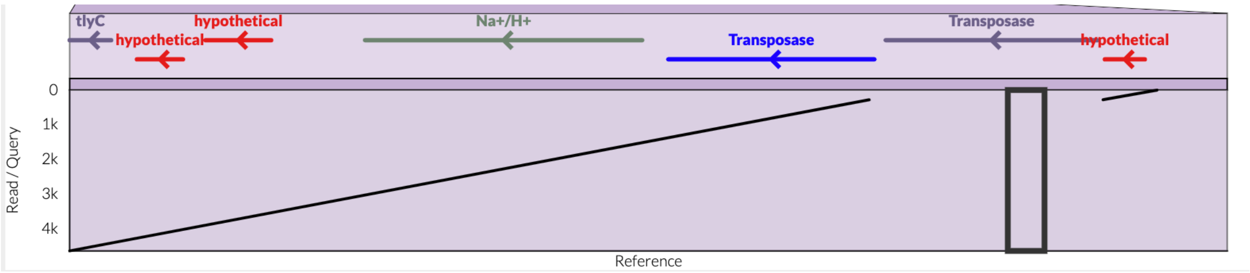

Supplemental Figure 6. Unitig_146 from the metagenomic assembly was identified as either a potential HGT event or mis-binning. When reassembled with the PB-SRB1 binned contigs alone, this sequence data was represented on a comparable contig, Unitig_26, which was in a complex region of the PB-SRB1 assembly group as shown in panel **(A)**, where each node represents a contig, and each edge an overlap (graph derived from the Celera best.edges file). In panel **(B)**, we see the origin of this complexity lies in structural variants associated with transposons (contig sequences in A were aligned and visualized with ProgressiveMauve).

A

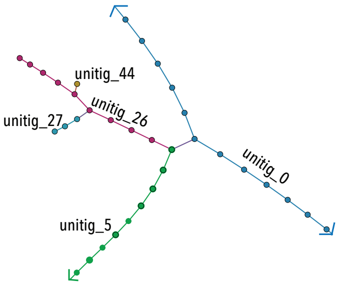

B

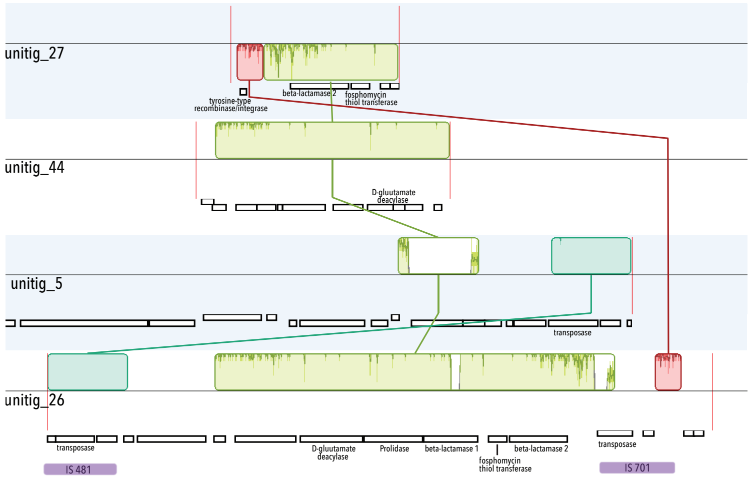

Supplemental Figure 7. (A) Unitig_102, which was linked to the *Micavibrionaceae* MAG based on methylation patterns, encodes a *Siphoviridae* dsDNA genome*,* with both structural and DNA replication genes. **(B)** Ten of unitig_102's coding sequences shared high percent identity (32-66% aa identity) with a cultured temperate phage, phiJI001, known to infect an alphaproteobacterial isolate from the genus *Labrenzia*, visualized here with progressiveMauve.

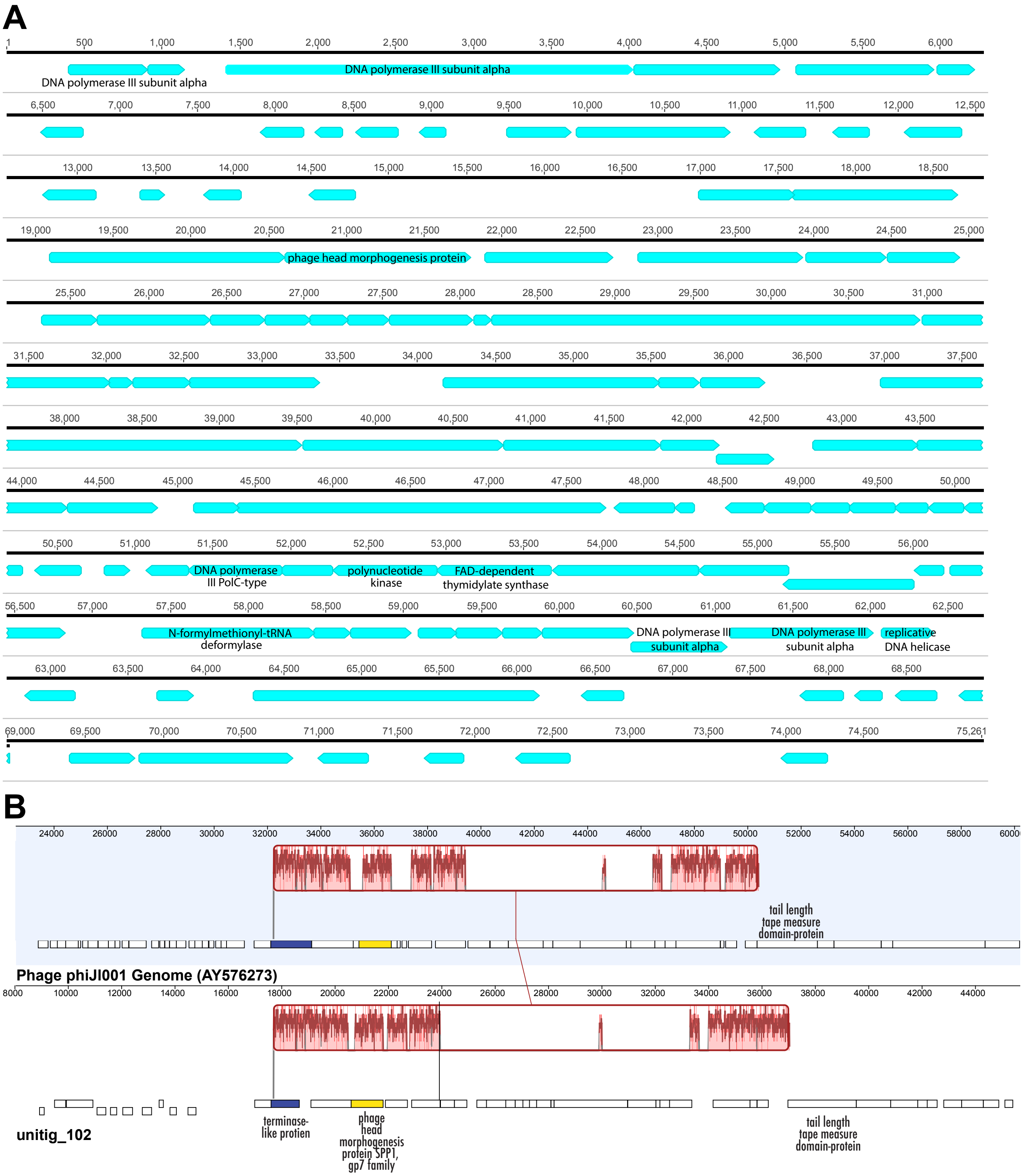

Supplemental Figure 8. Phylogenetic tree of 6-mA methyltransferases (MTases) that show a significant homology with *ccrM* from *Caulobacter crescentus* (blue), including the GANTC MTases from the pink berry alphaproteobacterial MAGs (shown in pink with IMG gene ID in brackets). Novel sequences were aligned using hmmalign to the reference alignment from Gonzalez et al. 2014, and the phylogeny was inferred with FastTree using gamma-distributed rates of site evolution and the WAG substitution matrix. Support values are shown at the nodes.

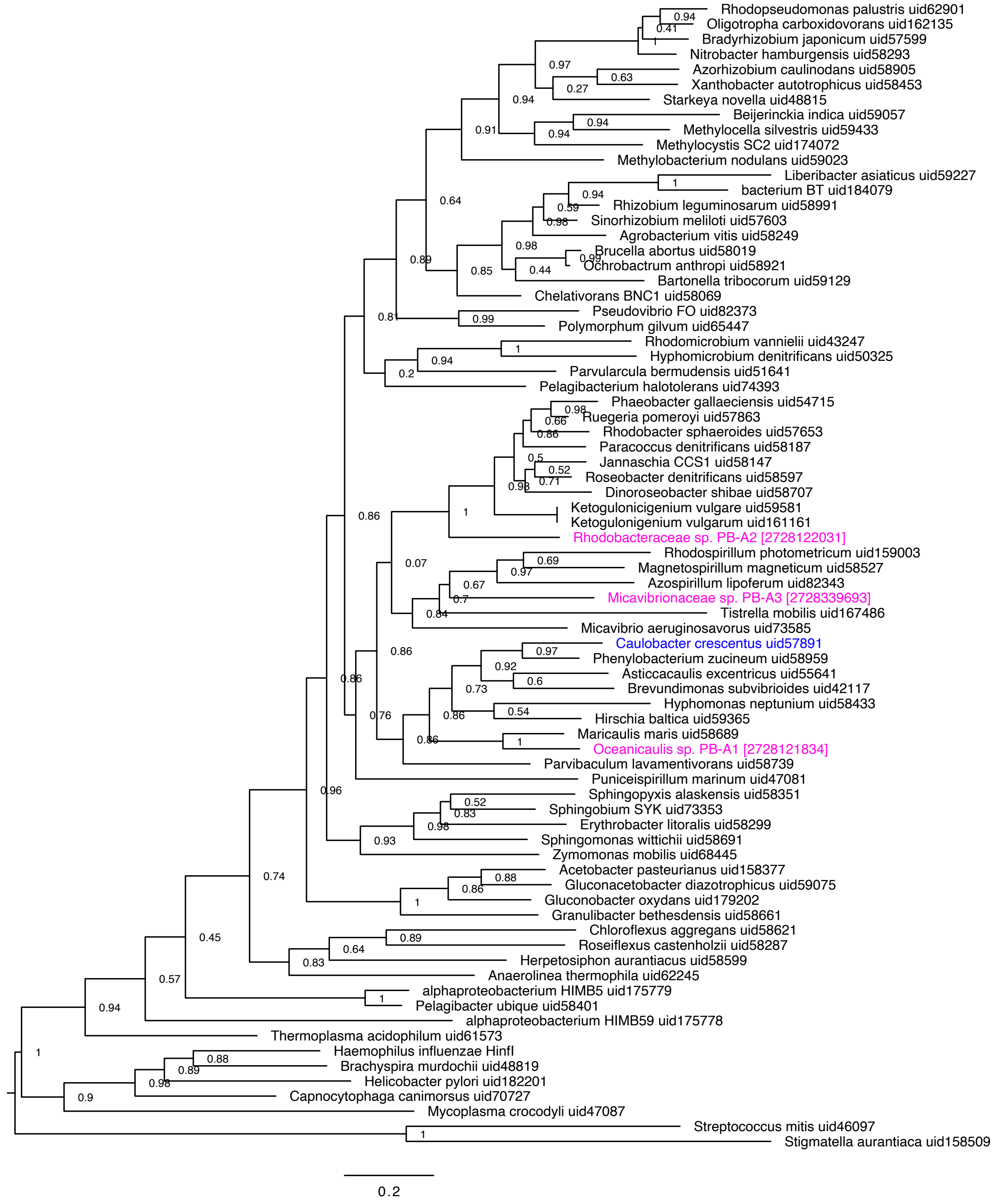

Supplemental Figure 9. *E. coli*'s *EcoprrI* **(A)** provides an example of an RM system with two different avenues to halt the spread of a phage infection, either a classic DNAse-based Type I RM defense **(B)** or an RNAse-based abortive infection strategy **(C)**. Encoded by a four gene operon **(A)**, the complex consists of PrrC, an $tRNA^{Lys}$-specific anticodon nuclease, associated with a typical Type I RM system (a methyltransferase (PrrA / HsdM, "M"), a specificity determinant that interacts with the DNA binding site (PrrB / SsdS, "S"), and a restriction enzyme (PrrD / HsdR, "R"). **(B)** EcoprrI assembles as a single protein complex, like other Type I RM systems, but with the addition of a latent, inactive PrrC subunit. During normal growth or infection by a susceptible virus, such as phage lambda, EcoprrI operates as a typical Type I restriction enzyme, and halts viral replication by cleaving the DNA of the infecting phage. **(C)** T4-phage encodes a resistance mechanism: a short peptide (Stp) which can bind to EcoprrI and inhibit its endonuclease activity, likely due to a conformational change. However, this same Stp-induced conformational change activates the complex's PrrC anticodon nuclease which cleaves host $tRNA^{Lys}$. This RNAse activity depletes the host's $tRNA^{Lys}$ which inhibits protein synthesis and kills the host. This "abortive infection" strategy, where the host cell detects resistant phage and sacrifices itself, stops viral replication and minimizes the spread of phage to the host's vulnerable clonal kin. Figure adapted from reference [53] of the maintext. We propose that the co-occurrence of Type I RM and other RNAse toxin genes in the *Thiohalocapsa* sp. PB-PSB1 genome could represent an analogous system, combining both RM and abortive infection phage defenses.

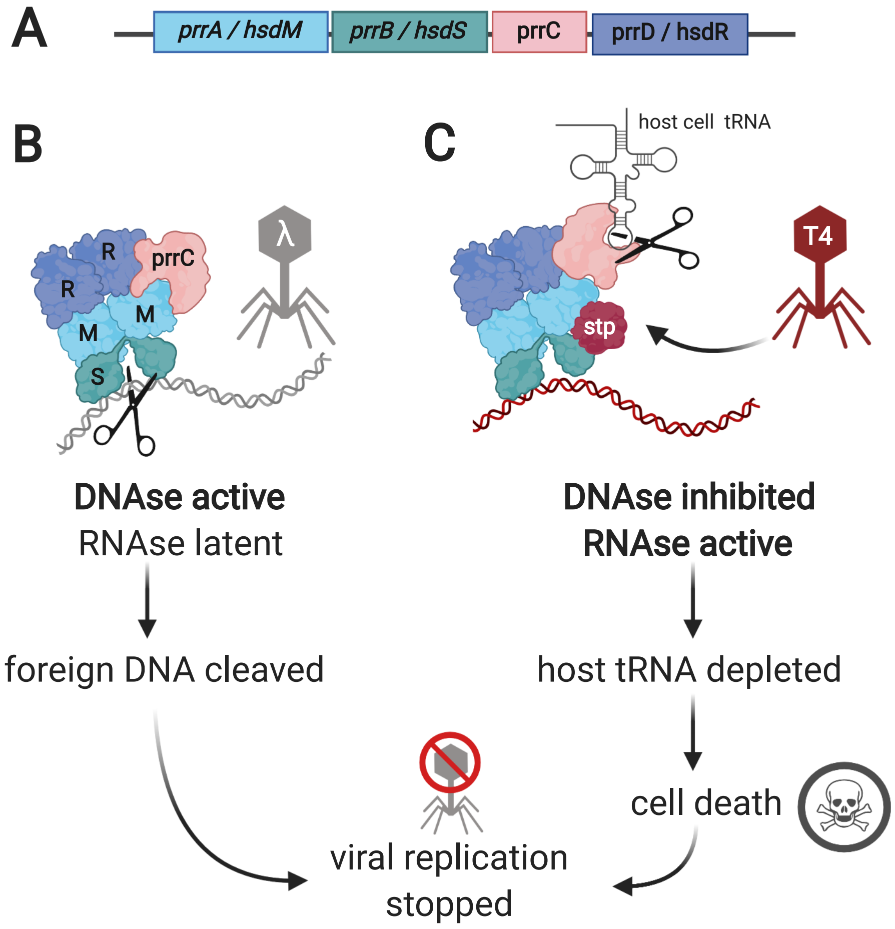

Supplemental Table 1. Statistics of the sequenced reads in the present study. Data shown for PacBio sequencing pertains to either from subreads passing quality filtering (filtered) or error-corrected subreads (error corrected). All sequence data are publicly available in NCBI SRA, associated with the provided NCBI BioSample identifier. Individual SRA accessions are listed in Supplemental Data 3.

Sequencing platform	Sample name	Sample description	NCBI BioSample	Read number	Read N50	Max length (bp)	Min length (bp)	Total size (Gb)	# of SMRT Cells
PacBio (filtered)	berry9	one large aggregate (~9 mm)	SAMN17053571	2,737,334	2,930	27,801	500	7.14	25
PacBio (filtered)	s01	13 pooled aggregates	SAMN17054265	154,600	6,102	27,043	500	0.58	17
PacBio (error corrected)	berry9 + s01	combined in silico	SAMN17228542	96,933	6,946	20,798	500	0.47	42
Illumina MiSeq (250 PE)	s02	10 pooled aggregates	SAMN17054266	26,384,468	250	250	250	6.60	
Illumina HiSeq (150 PE)	s02	10 pooled aggregates	SAMN17054266	357,155,148	150	150	150	53.57	
Illumina MiSeq (250 PE)	berry9	one large aggregate (~9 mm)	SAMN17053571	4,744,750	250	250	250	1.19	

Supplemental Table 2. Statistics of the assembled sequence data. NCBI accessions are listed in Supplemental Data 3.

Sequencing platform	Sample name	NCBI BioSample	Assembler	# contigs	N50	Max length (bp)	Min length (bp)	Total size (Mb)
PacBio	berry9 + s01	SAMN17228542	HGAP3	169	412,938	3,494,615	8,751	18
Illumina	s02 (HiSeq + MiSeq)	SAMN17054266	idba_ud	35,372	8,379	521,863	1,000	127

Supplemental Table 3. 6mA and 4mC modified sequence motifs identified from large contigs in the assembled pacbio data using the SMRT Analysis workflow. The modification profiles on each contig for these 32 motifs, m01 – m32, are shown in Figure 2 (names correspond to those shown in Figure 2).

Motif ID	Motif sequence
m01	GAGNNNNNNAATC
m02	GATTNNNNNNCTC
m03	CTGCAG
m04	CTCGAG
m05	CTACKAC
m06	AAGCTT
m07	CCTNAGG
m08	CCCACA
m09	ACCCAG
m10	GCGCGAT
m11	GAGATG
m12	AGGGCC
m13	CCANNNNNNGTCA
m14	TGACNNNNNNTGG
m15	AGGCCT
m16	GCANNNNNNTCAC
m17	GTGANNNNNNTGC
m18	AYGCCGC
m19	GGATCC
m20	GCTGAT
m21	GCANNNNNNNGTTG
m22	CAACNNNNNNNTGC
m23	CGCGA
m24	CGCANNNNNNGGG
m25	CCAGCG
m26	GGWCC
m27	GAYCCC
m28	RGATCY
m29	GANTC
m30	CANCATC
m31	GCCAGG
m32	GATGGA

Supplemental Table 4. The 7 bins recovered by metabat2 corresponded to individual methylation groups (abbreviated MG, defined in main text Figure 2). Contaminating contigs in these MetaBAT2 bins were defined as those originating from a different methylation group. The percentage of the total methylation group sequence (bp) recovered by MetaBAT2 was calculated (=100*size metabat2 bin / size of methylation group). Where two MetaBAT2 bins corresponded to one methylation group these were combined to calculate the combined recovery.

MetaBAT2 bin ID	# contigs	Size (bp)	Methylation group	Taxonomic assignment	CheckM completeness	CheckM contamination	Contaminating contigs (#)	% of MG sequence	Combined % of MG sequence
1	4	438,011	1	*Desulfofustis* sp. PB-SRB1	21%	0%	0	11%	95%
4	23	3,460,210	1	*Desulfofustis* sp. PB-SRB1	74%	0%	0	84%	
2	16	3,863,393	5	*Rhodobacteraceae*	92%	1%	0	81%	86%
7	5	236,158	5	*Rhodobacteraceae*	0%	0%	0	5%	
3	4	1,254,053	7	*Thiohalocapsa* sp. PB-PSB1	22%	0%	0	15%	96%
6	11	6,796,591	7	*Thiohalocapsa* sp. PB-PSB1	78%	2%	0	81%	
5	15	250,306	3	*Oceanicaulis alexandrii*	2%	0%	0	41%	
low depth	7	96,964							
unbinned	84	1,618,953							

Supplemental Table 5. MaxBin recovered 3 bins, two of which were highly contaminated, containing sequence from multiple different organisms. Column definitions are as described for Supplemental Table 4.

MaxBin bin ID	# contigs	Size (bp)	Methylation group	Taxonomic assignment	CheckM completeness	CheckM contamination	Contaminating contigs (#)	% of MG sequence
1	16	8,065,021	7	*Thiohalocapsa* sp. PB-PSB1	99%	4%	0	97%
2	88	5,423,509	2,3,4,5,7	mixed *Alphaproteobacteria*	96%	26%	40	NA
3	65	4,526,109	1,2,3,4,6,7	*Desulfofustis* sp. PB-SRB1	99%	5%	26	NA

Supplemental Reference:

1. Dojka MA, Hugenholtz P, Haack SK, Pace NR. Microbial diversity in a hydrocarbon- and chlorinated-solvent-contaminated aquifer undergoing intrinsic bioremediation. *Appl Environ Microbiol*. 1998; **64**(10): 3869-77.
2. Seemann T. Prokka: rapid prokaryotic genome annotation. *Bioinformatics*. 2014; **30**(14): 2068-9.
3. Altschul SF, Madden TL, Schaffer AA, Zhang JH, Zhang Z, Miller W, et al. Gapped BLAST and PSI-BLAST: a new generation of protein database search programs. *Nucleic Acids Res*. 1997; **25**(17): 3389-402.
4. Huson DH, Mitra S, Ruscheweyh HJ, Weber N, Schuster SC. Integrative analysis of environmental sequences using MEGAN4. *Genome Res*. 2011; **21**(9): 1552-60.
5. Wu D, Jospin G, Eisen JA. Systematic identification of gene families for use as "markers" for phylogenetic and phylogeny-driven ecological studies of bacteria and archaea and their major subgroups. *PLoS One*. 2013; **8**(10): e77033.
6. Parks DH, Imelfort M, Skennerton CT, Hugenholtz P, Tyson GW. CheckM: assessing the quality of microbial genomes recovered from isolates, single cells, and metagenomes. *Genome Res*. 2015; **25**(7): 1043-55.
7. Yoon SH, Ha SM, Lim J, Kwon S, Chun J. A large-scale evaluation of algorithms to calculate average nucleotide identity. *Anton Leeuw Int J G*. 2017; **110**(10): 1281-6.
8. Tatusova T, DiCuccio M, Badretdin A, Chetvernin V, Nawrocki EP, Zaslavsky L, et al. NCBI prokaryotic genome annotation pipeline. *Nucleic Acids Res*. 2016; **44**(14): 6614-24.
9. Xie ZQ, Tang HX. ISEScan: automated identification of insertion sequence elements in prokaryotic genomes. *Bioinformatics*. 2017; **33**(21): 3340-7.
10. Akhter S, Aziz RK, Edwards RA. PhiSpy: a novel algorithm for finding prophages in bacterial genomes that combines similarity- and composition-based strategies. *Nucleic Acids Res*. 2012; **40**(16): e126.
11. Guo JR, Bolduc B, Zayed AA, Varsani A, Dominguez-Huerta G, Delmont TO, et al. VirSorter2: a multi-classifier, expert-guided approach to detect diverse DNA and RNA viruses. *Microbiome*. 2021; **9**(1): 37.
12. Alcock BP, Raphenya AR, Lau TTY, Tsang KK, Bouchard M, Edalatmand A, et al. CARD 2020: antibiotic resistome surveillance with the comprehensive antibiotic resistance database. *Nucleic Acids Res*. 2020; **48**(D1): D517-D25.
13. Sedlazeck FJ, Rescheneder P, Smolka M, Fang H, Nattestad M, von Haeseler A, et al. Accurate detection of complex structural variations using single-molecule sequencing. *Nature Methods*. 2018; **15**(6): 461-8.
14. Thorvaldsdottir H, Robinson JT, Mesirov JP. Integrative Genomics Viewer (IGV): high-performance genomics data visualization and exploration. *Brief Bioinform*. 2013; **14**(2): 178-92.
15. Nattestad M, Aboukhalil R, Chin CS, Schatz MC. Ribbon: Intuitive visualization for complex genomic variation. *Bioinformatics*. 2020; **37**(3): 413-7.
