# Supplementary figures and images for: Metagenomic methylation patterns resolve bacterial genomes of unusual size and structural complexity
Source: ISME J. 2022 Apr 22;16(8):1921–31. doi: 10.1038/s41396-022-01242-7 (PMC9296519; doi:10.1038/s41396-022-01242-7)

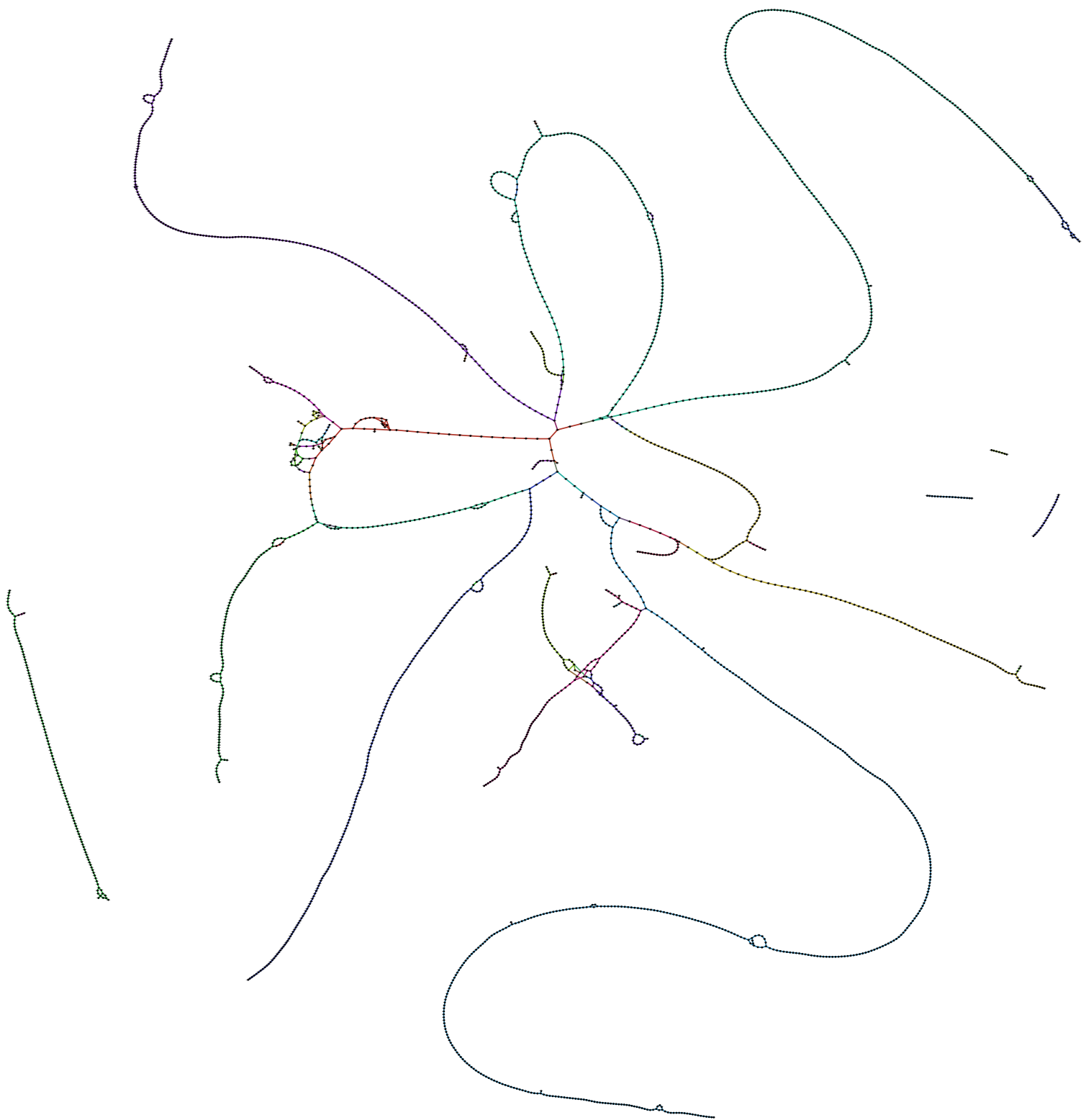

Supplement: Supplementary file 2 — Supplementary Data 4 [file 41396_2022_1242_MOESM2_ESM.zip › supplemental_data_4/SRB-asm-graph/PBSRB1_reasm.pdf]

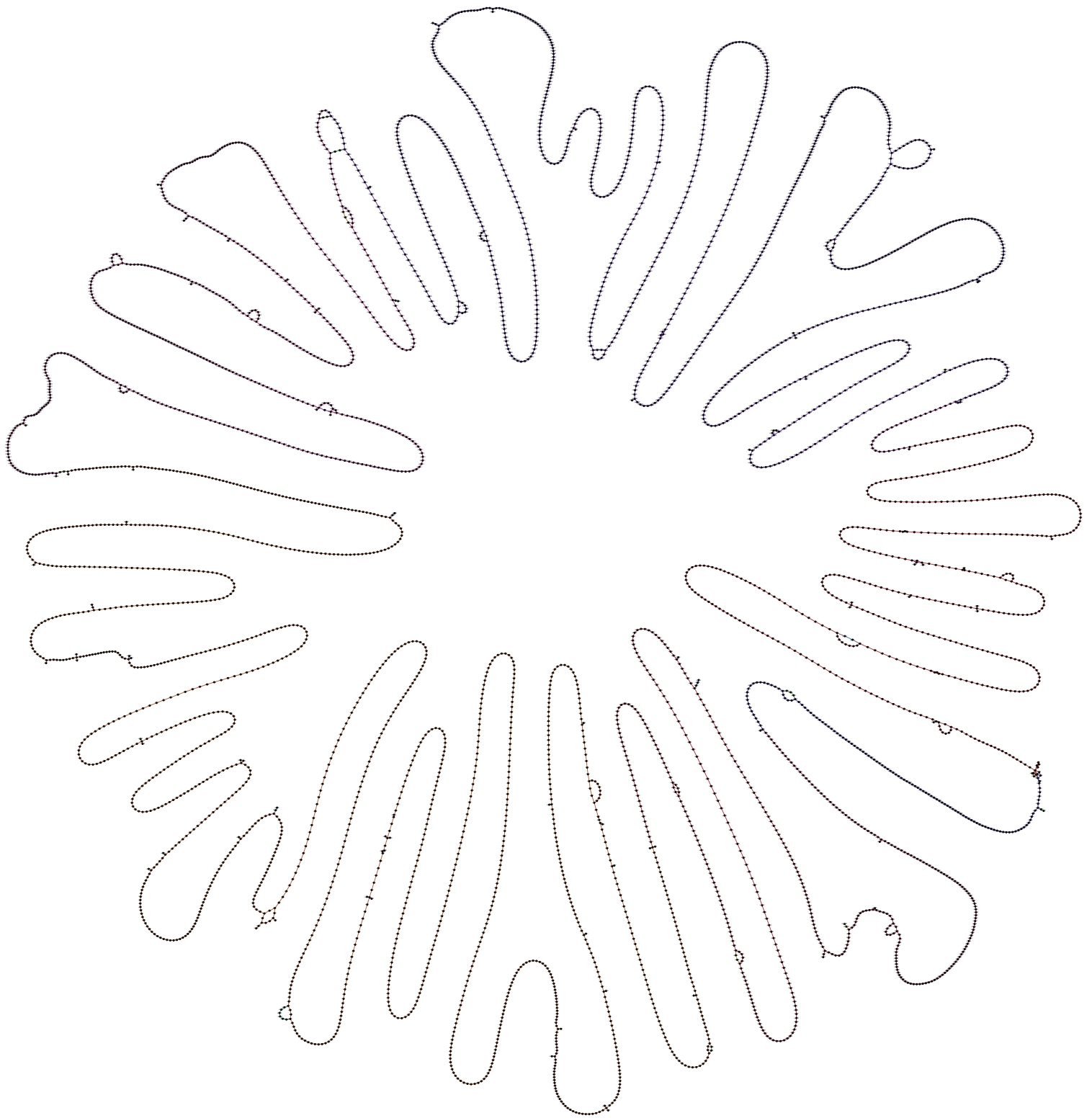

Supplement: Supplementary file 2 — Supplementary Data 4 [file 41396_2022_1242_MOESM2_ESM.zip › supplemental_data_4/PSB-asm-graph/PB-PSB1_reasm.pdf]

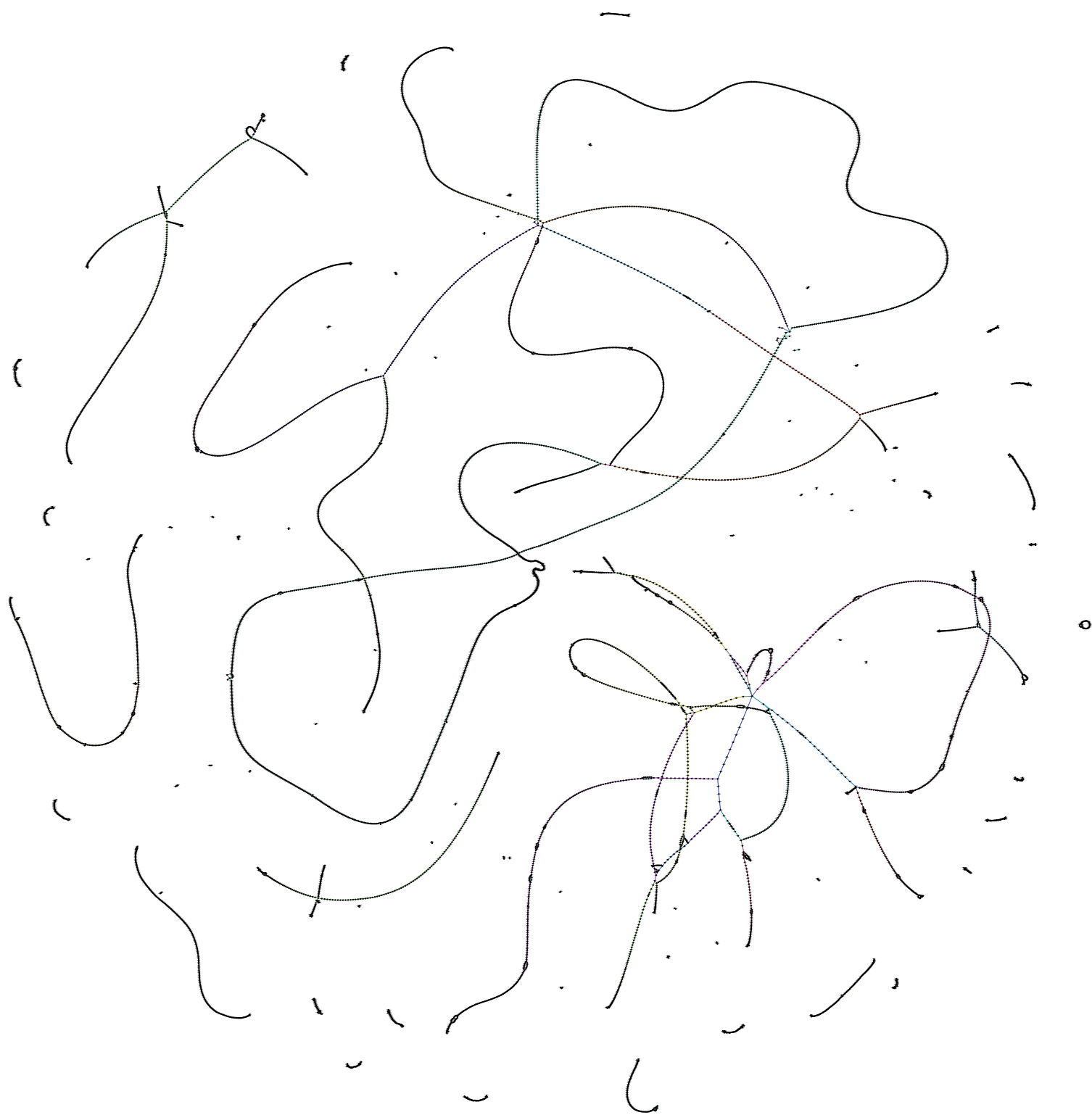

Supplement: Supplementary file 2 — Supplementary Data 4 [file 41396_2022_1242_MOESM2_ESM.zip › supplemental_data_4/metagenomic-asm/7771_asm-graph-gephi.pdf]

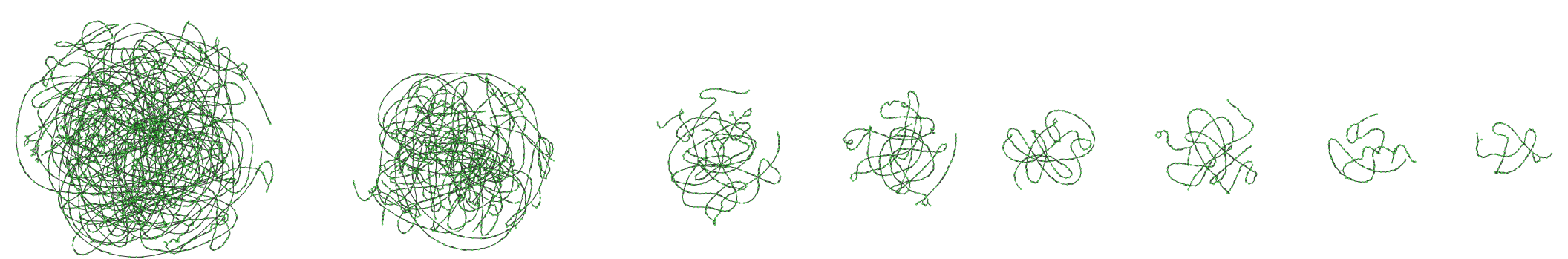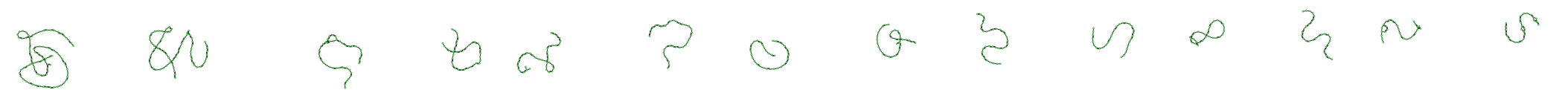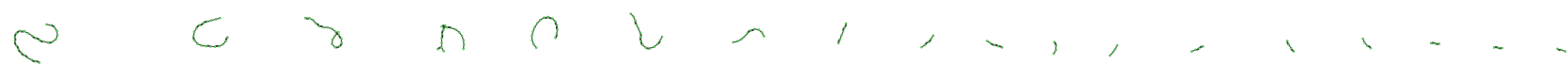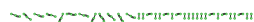

Supplement: Supplementary file 2 — Supplementary Data 4 [file 41396_2022_1242_MOESM2_ESM.zip › supplemental_data_4/metagenomic-asm/7771_asm-graph-cys.pdf]
